# Wild Bee Conservation: Habitat Heterogeneity and Ecological Improvement in Agricultural Land With Open Grassland

**DOI:** 10.1002/ece3.74514

**Published:** 2026-10-16

**Authors:** Julia Lanner, Pauline Bühler, Loïc Hellers, Timotheus Kopf, Alexander Weigand, Birgit C. Schlick‐Steiner, Florian M. Steiner

**Affiliations:** ^1^ Department of Ecology University of Innsbruck Innsbruck Austria; ^2^ Department of Integrative Biology, Institute of Zoology Boku University Vienna Austria; ^3^ Syndicat intercommunal à vocation multiple (SIAS) Niederanven Luxembourg; ^4^ National Museum of Natural History of Luxembourg Luxembourg City Luxembourg

**Keywords:** Apidae, Central Europe, community ecology, land use, nature conservation

## Abstract

Wild bee populations are declining due to habitat loss and degradation. Flower‐rich heterogenous grasslands have the potential to compensate for the loss and provide valuable habitat for wild bees. To investigate the effects of different levels of habitat heterogeneity and of ecological habitat improvement to increase foraging resources on wild bee communities, we comparatively studied two Central European grassland types in Austria (AT) and Luxembourg (LU) using pan traps. We collected and identified > 5000 individuals representing considerable fractions of wild bee fauna of these countries and characterised habitat heterogeneity (number of habitat elements, ecotone lengths) using GIS data and flower‐colour abundance (white, yellow, blue) using drone imaging. Our findings suggest that macro‐scale heterogeneity alone may not drive wild bee assemblages in LU, pointing to the potential overriding importance of unmeasured microhabitat variables, such as nesting substrates and taxonomic floral composition. In AT, however, wild bee abundance increased with habitat heterogeneity as measured by the length of ecotones with trees and bushes. Ecological improvement of habitat was important in LU, in that formerly intensively farmed land with sown wildflower fallows hosted higher richness and abundance than extensively used grassland. In AT, in contrast, higher species richness and abundance occurred in extensively used grassland than in formerly intensively farmed land with wildflower strips; here, overall increased floral resource abundance on ecologically improved sites seemed to have less influence, and flower species diversity, the quality of floral resources or any combination of flower properties, may have been more important than just its quantity as assessed by flower‐colour abundance. In conclusion, we emphasise the relevance of regional habitat heterogeneity to wild bees and the opportunities by ecological improvement of intensively farmed land. Conservation strategies should be tailored to regional landscape contexts, prioritising extensive grassland protection in Austria and targeted wildflower fallow establishment in Luxembourg.

## Graphical abstract

Here, we investigated the effects of different levels of habitat heterogeneity and of ecological improvement of habitat to increase foraging resources on wild bee communities in Austria and Luxembourg. We found that ecological improvement was important in Luxembourg for species richness and abundance but less so in Austria; here, habitat heterogeneity was important as was the quality of flowers rather than sheer quantity. Thus, our findings illustrate the chances opened up by biodiversity improvement measures but at the same time emphasise the need for conservation of lowland grassland habitats for wild bee diversity.

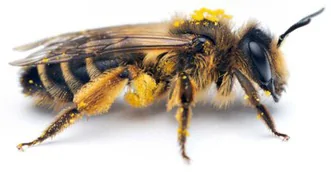

## 1 Introduction

Bees are among the most important pollinators of angiosperms, economically and ecologically (Klein et al. 2007; Ollerton et al. 2011). Stable wild bee populations require adequate nesting grounds, nest building material and foraging resources consisting of nectar and pollen to fulfil their life cycles (Figure 1). Suitable wild‐bee habitat must provide these resources in a certain quantity and quality within species‐specific flight distances of mostly a few hundred metres (Zurbuchen, Cheesman, et al. 2010; Zurbuchen, Landert, et al. 2010; Westrich 2018). Thus, a high heterogeneity of environmental structures and the availability of diversified communities of flowering plants define habitats with the highest bee diversity and abundance (Steffan‐Dewenter et al. 2002; Potts et al. 2003).

**FIGURE 1:**
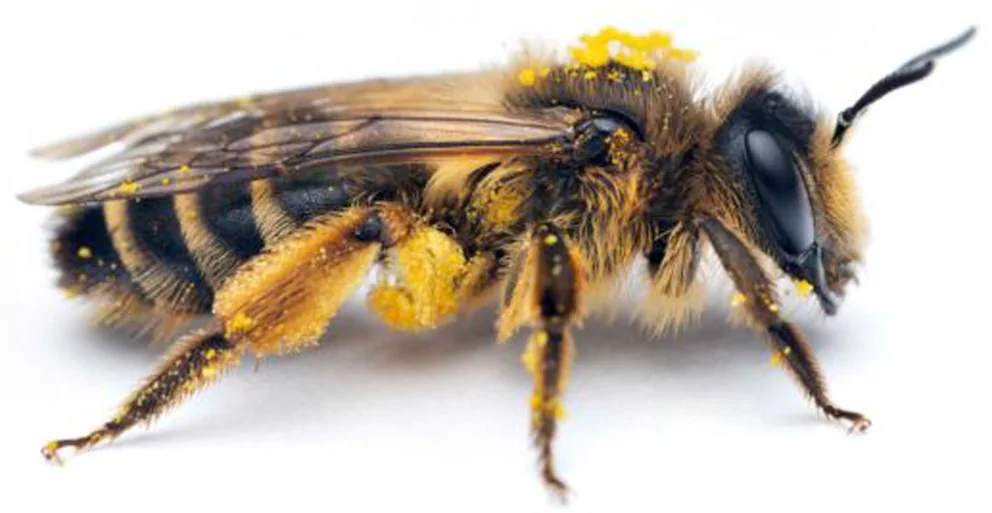
A female of the yellow‐legged mining bee *Andrena flavipes* Panzer, 1799. The species is a polylectic forager and creates self‐dug nests in sandy soils. Photo: Beelibre.lu (do Rosário Cruz et al. 2025).

One of the defining elements of landscapes is ecotones, that is, transitional areas between habitat types, which can have different impacts on species based on their particular natural history (Olynyk et al. 2021). For example, in agriculturally managed landscapes, hedgerows and forest edges are particularly important for pollinators offering diverse soil microhabitats for nesting (Kells and Goulson 2003; Hofmeister et al. 2019; Martin et al. 2019), while wildflower strips within cultivated areas lead to a higher diversity and abundance of wild bees (Haaland et al. 2011).

However, environmental changes, including habitat loss, climate change, arrival of invasive species and pathogens, as well as exposure to pesticides, pose a threat to the survival of wild bee populations, leading to a global decline of species numbers and abundance (Brown and Paxton 2009; Potts et al. 2010; Hallmann et al. 2017; Zattara and Aizen 2021). Habitat fragmentation and degradation together with intensive land management have been revealed as the major drivers of agricultural landscape change and explanatory factors for a declining diversity of bees (Winfree et al. 2009; Ollerton et al. 2014; LeBuhn and Vargas Luna 2021). Intensive cultivation of agricultural land often leads to the loss of structures and flower resources, resulting in barren landscapes for wild bee and other insect populations (Kremen et al. 2002; Foley et al. 2005; Sánchez‐Bayo and Wyckhuys 2019). Subsequently, the loss of structures in agricultural landscapes can cause a spatial dissociation between foraging and nesting areas, and wild bees, particularly specialised species with small flight ranges and univoltine life cycles, may experience population declines or local extinction (Biesmeijer 2006; Zurbuchen and Müller 2012).

To halt or even reverse the trend of declining wild bee populations in homogeneous and flower‐poor landscapes, conservation efforts in agriculture and land management have become more and more important (Klein et al. 2007; Winfree 2010). For example, lowland hay meadows are classified as special protection areas under the European Habitat Directive (Council Directive 92/43/EEC; habitat type 6510) due to their semi‐natural, extensively farmed habitats comprising diverse flowering plant communities that offer exceptional foraging opportunities for a range of pollinators (Ziaja et al. 2018). In addition to legal protection, implementing low‐intensity mowing and cultivating species‐rich wildflower strips within farmland could help counteract anthropogenic pressures on European meadows and prevent declines in wild pollinator populations (Steffan‐Dewenter et al. 2002; Kosior et al. 2007). To assess the potential for pollinators and safeguard high‐quality habitats and resources, it is essential to identify and subsequently monitor protected habitats and ecologically improved areas (Walz 2011; Ziaja et al. 2018; Breeze et al. 2021).

In this study, we comparatively analysed wild bee communities in Central Europe (Austria, AT and Luxembourg, LU) in two grassland habitat types: grasslands with extensive land‐use management regimes (extensively farmed, EF_AT and EF_LU) as sites expected to be regionally optimal by the authors for wild bees at the onset of the study, that is, considered as benchmarks, and formerly intensively farmed land, on which management intensity had been relaxed and improvement measurements regionally available to farmers had been applied (intensively farmed; IF_AT, meadows with a wildflower strip on one margin that is mown just once a year; IF_LU, sown wildflower fallows). Both in AT and LU, the level of habitat heterogeneity—the structural variation of a habitat across space—differed considerably within both habitat types. Based on the reported relevance of habitat heterogeneity to wild bee communities (Banaszak and Szefer 2014; Sobieraj‐Betlińska et al. 2023), we expected that (i) more heterogeneous habitats host higher wild bee abundance, species richness and biomass than less heterogeneous ones. This can be assumed because small‐bodied wild bee species, in particular, need the spatial co‐occurrence of their preferred floral resources, nesting materials and nesting grounds—which is more likely in heterogeneous habitats—as they have limited capabilities to overcome large flight distances (Greenleaf et al. 2007); in testing this, we used ecotone quantifications of each site as proxies for overall habitat heterogeneity in the sense of landscape composition and patch complexity. Further, as lowland hay meadows are known for their rare and diverse plant species (Ziaja et al. 2018) and since generalist flower visitors and large‐bodied wild bee species are more likely to overcome greater foraging distances (Peterson and Roitberg 2006; Zurbuchen, Cheesman, et al. 2010; Zurbuchen, Landert, et al. 2010), we hypothesised that (ii) extensively used grassland (EF_AT, EF_LU) hosts higher wild bee species richness and diversity in ecological guilds with many oligolectic species but hosts a lower biomass compared with ecologically improved formerly intensively farmed land (IF_AT, IF_LU) that includes wildflower strips (IF_AT) or wildflower fallows (IF_LU) and thus attracts more generalist flower visitors and large‐bodied species; in testing this, we also considered ecotone quantifications as well as the cover of white, yellow and blue flowers on each site, given that floral colours are important for attracting pollinators (Binkenstein et al. 2013, and references therein). Finally, and based on the results of our comparative study, we considered more generally conservation measurements for grassland in Central Europe.

## 2 Material and Methods

### 2.1 Study Sites

Each habitat type was represented in both regions, western AT (Tyrol) and southern LU, the latter spanning parts of the cantons Esch‐sur‐Alzette, Luxembourg and Remich. A total of 680 wild bee species have been documented in AT (Reverté et al. 2023), with an estimated 400 species in Tyrol (T. Kopf, unpublished), and a total of 346 wild bee species in LU (Herrera Mesías and Weigand 2021). In each region, 14 study sites were selected and categorised according to their farming intensity, that is, seven extensively farmed (EF) and seven ecologically improved (formerly) intensively farmed (IF) sites per region.

For the AT study area, representative average temperature/cumulative precipitation (Innsbruck, Airport; https://www.zamg.ac.at/fix/klima/oe71‐00/klima2000/daten/klimadaten/tir/11800.htm) were for April 2021, May 2021, June 2021, July 2021 and the whole year 2021 8.4°C/57.7 mm, 13.4°C/87.1 mm, 16.1°C/110.3 mm, 18.1°C/137.2 mm and 8.5°/896.5 mm, respectively. The EF_AT sites were seminatural habitat, which was extensively managed due to unfavourable farming of hillsides, wetlands or recreational use (Figure 2, Figure S1), whereas IF_AT sites were under intensive management regime, among which some extensively managed meadows are scattered as the whole habitat complex is part of a Natura 2000 protected area (Ortolan—Silz—Haiming—Stams). Each IF_AT site included one flowering strip of 2 m width each. These wildflower strips differed from the meadows they were part of only in their mowing regime—they were mowed only once a year, in the course of the meadow's second mowing event in August—but no seeding took place (i.e., the entire meadows including the flower strips were covered by spontaneous vegetation).

**FIGURE 2:**
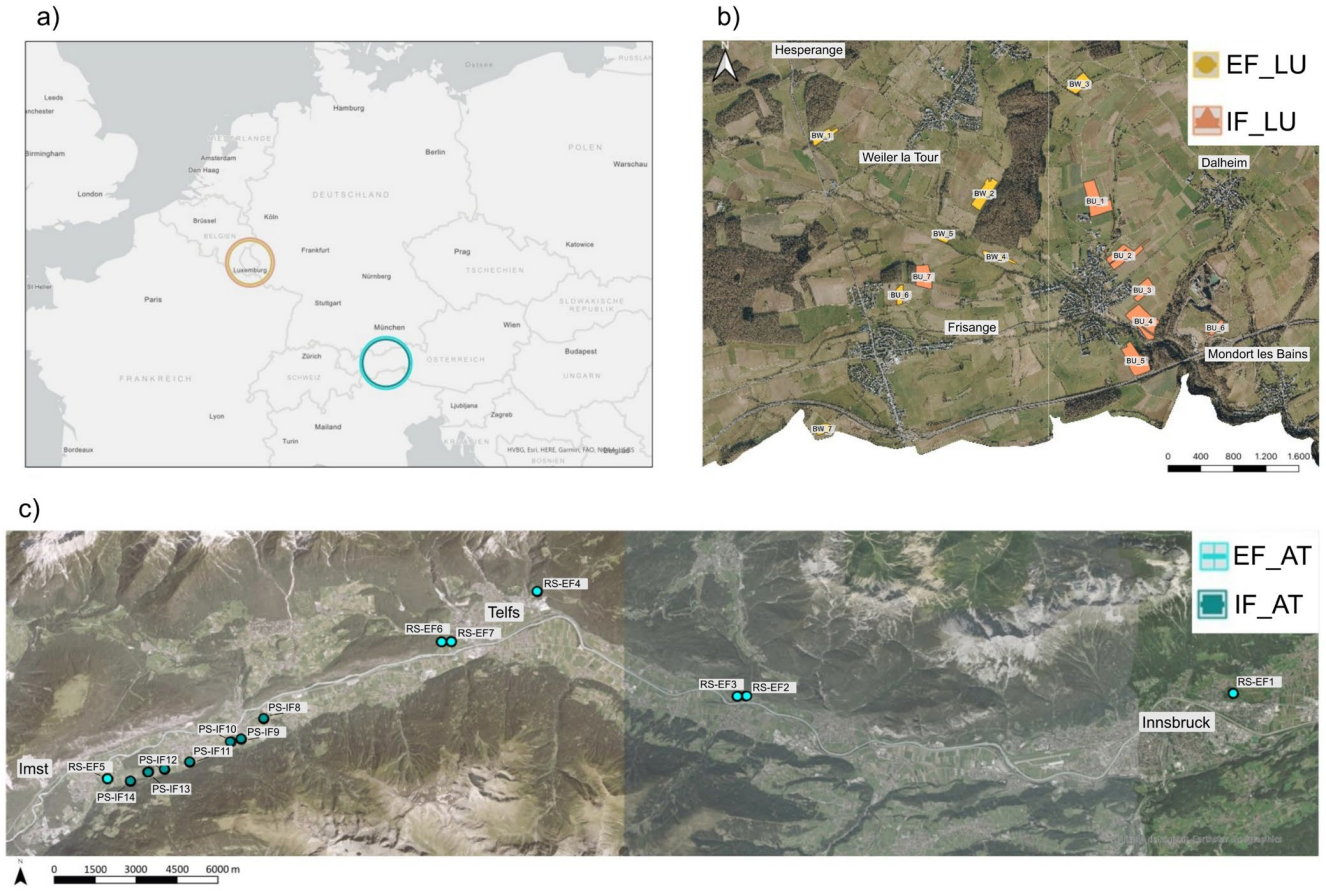
(a) The study was conducted in two countries in Central Europe, in Austria (blue) and in Luxembourg (orange). (b) The 14 study sites in Luxembourg were located in the south of the country, in the municipality of Fréiseng, Weiler‐la‐Tour and Mondorf‐les‐Bains (EF_LU = extensively used grassland, BW1 to BW7; IF_LU = ecologically improved formerly intensively farmed land, BU1 to BU7). (c) The 14 study sites in Austria were located in the west of the country, in the federal state of Tyrol (EF_AT = extensively used grassland, RS‐EF1 to RS‐EF7; IF_AT = ecologically improved formerly intensively farmed land, PS‐IF8 to PS‐IF14).

For the LU study area, representative average temperature/cumulative precipitation (Luxembourg, Findel; https://lustat.statec.lu/vis?lc=en&tm=DF_A2100&pg=0&snb=1&df[ds]=ds‐release&df[id]=DF_A2100&df[ag]=LU1&df[vs]=1.1&dq=A.L01.LU0000304_FINDEL&pd.=%2C2025&to[TIME_PERIOD]=false) were for April 2021, May 2021, June 2021, July 2021 and the whole year 2021 6.7°C/36.2 mm, 10.0°C/80.8 mm, 8.7°C/55.8 mm, 17.2°C/187.8 mm and 9.4°/784.2 mm, respectively. The LU study sites comprised seven legally protected lowland hay meadows of the Habitats Directive habitat type 6510 (EF_LU habitats), of which two were situated in the Natura 2000 area of Aspelt, Lannebuer am Kassel, and seven recently cultivated wildflower fallows on formerly intensively farmed agricultural land (IF_LU habitats) (Figure 2, Figure S1). All IF_LU habitats and five EF_LU habitats were subject to biodiversity contracts in accordance with the national law of the ‘Grand‐Ducal Regulation of 11 September 2017 establishing a set of aid schemes for safeguarding biological diversity in rural areas’ (translated to English by the authors). Wildflower fallows were seeded by using the seed mixture of Rieger‐Hoffmann GmbH (dominant flowering plants and seed mixture are indicated in Tables S1 and S2) and bloomed for the first time in 2019 or 2020. No management was applied to the IF_LU sites, whereas EF_LU sites were either mowed twice a year before 15 June or 1–2 times after 15 June.

### 2.2 Wild Bee Sampling and Identification

We collected bees by using pan traps following recommendations set out by Potts et al. (2020). Ideally, and considering that each sampling method has its strengths and weaknesses, a combination of sampling methods should be applied for comprehensively assessing communities; this, however, would have been impractical here due to the increased logistic effort. We therefore opted for pan traps in three colours, which also are effective in catching high species numbers (Joshi et al. 2015) and are widely applied in bee community ecology studies (see review of Krahner et al. 2024). Limitations of this sampling setup are outlined in the discussion section.

In more detail, each set of pan traps included three coloured bowls (white, RAL [*Reichs‐Ausschuß für Lieferbedingungen und Gütesicherung*] value 9010; yellow, 1021; blue, 5010; Malerversand FPT‐Group, Teningen, Germany) with a capacity of 800 mL filled with salty water (180 g NaCl/L) and odourless detergent to reduce surface tension, keeping bees submerged in the trap. We positioned the pan trap sets 5–10 cm below the top of 120 cm long wooden sticks (Figure S2), which in turn were entered approx. 30 cm into the soil. This resulted in pan traps being approx. 80–90 cm above ground and slightly above natural vegetation. At each site, three pan trap sets were installed at each site (3 pan trap sets × 14 sites = 126 pan traps per region) with a minimum distance of 15 m between the wooden sticks respecting minimum trap distance requirements as suggested by Krahner et al. (2024). We emptied and refilled each pan trap on average every 10.1 days (±3.0; min. 6 days due to mowing actions; max. 17 days due to bad weather conditions) during eight sampling periods (T1–T8). During particularly hot and dry periods, salt content was increased to avoid complete evaporation of the fixative. To cope with particularly rainy periods and prevent the samples from spilling over, four tiny holes were drilled close to the opening of each pan trap bowl to allow the water to drain away. After initial field visits at IF_LU sites, it became apparent that two preventative measures needed to be installed to protect against damage caused by wildlife: (1) green robes and nails have been attached to the top of each wooden stick at sites IF_LU plots 1 and 2 to prevent birds scavenging the caught but largely submerged bees, in particular ravens which showed a high local density and (2) tights stuffed with dog hair have been added to wooden sticks at all IF‐LU sites to olfactorily distract wild boars from visits and potentially destroying the experimental setup.

A 3‐month sampling period spanning mid‐April to mid‐July 2021 was chosen to cover the main activity periods of spring‐ and early‐summer‐flying wild bee species, while generating a logistically still manageable sampling effort but mostly neglecting late‐summer‐ and autumn‐flying species (in particular for abundance data and inferred biomass estimates). Howsoever, strictly late‐flying wild bee species only make up the minority of all wild bee species in the two target regions, and several species are not expected to occur on the investigated plots due to the absence of their specialised floral resources, such as *Colletes hederae* on common ivy, *Colletes succinctus* on Ericaceae and *Melitta tricincta* on *Odontites*.

The collected wild bees were pre‐sorted from the bulk samples in the laboratory and morphologically identified to the species level using the keys of Scheuchl (1995), Amiet (1996, 2001), Schmid‐Egger and Scheuchl (1997), Amiet et al. (2004) and Scheuchl and Willner (2016). The nomenclature of Ghisbain et al. (2023) was followed. Honeybee individuals were counted (EF_AT = 1805; IF_AT = 826; EF_LU = 570; IF_LU = 511; Tables S3 and S4, Figure S3) but excluded from the data set for further analyses. Wild bee collection material was stored at the University of Innsbruck in a 70% ethanol solution and in the National Museum of Natural History of Luxembourg (MNHNL) dry (pinned) and wet collection (96% ethanol). Other taxa and honeybees were transferred either to the Tyrolean Museum of Nature (Tiroler Landesmuseum, Ferdinandeum Innsbruck) or the MNHNL as well. Specimens that could not be morphologically unambiguously identified to species level, or specimens for which another line of taxonomic evidence was needed (e.g., potential first records in a country), were DNA barcoded at the MNHNL using the protocol of Weigand and Herrera‐Mesías (2020): *Andrena cineraria* / *A. vaga* (*n* = 7), *Andrena nigroolivacea* (*n* = 1), *Andrena tibialis* (*n* = 1), *Andrena wilkella* / *A. ovatula* / *A. dorsata* (*n* = 5), *Bombus hortorum* / *B. ruderatus* (*n* = 2), *Bombus lapidarius* / *B. ruderarius* (*n* = 5), *Bombus terrestris* / *B. lucorum* (*n* = 89), *Lasioglossum angusticeps* (*n* = 1), *Lasioglossum nitidulum* / *L. morio* (*n* = 1), *Seladonia leucahenea* (*n* = 1) (Data S5, including nucleotide sequences). Since not all targeted specimens yielded sufficient DNA quantity and/or quality and since we thus failed to generate DNA barcodes for all specimens of a taxonomic grouping, some taxa had to be dealt with at higher taxonomic levels for consistency purposes, that is, *Andrena ovatula* ‐complex, *Halictus simplex* ‐complex as well as some *Bombus* spp. (Table 1).

**TABLE 1:** Bee taxa recorded in this study in Austria (AT) and Luxembourg (LU), identified at the finest taxonomic resolution feasible (species, species complex or genus). Taxa that were genetically validated are marked with an asterisk (*).

Taxon	Author, Year	AT	LU
*Andrena alfkenella*	Perkins, 1914	x	x
*Andrena barbilabris*	(Kirby, 1802)		x
*Andrena bicolor*	Fabricius, 1775	x	x
*Andrena chrysosceles*	(Kirby, 1802)		x
Andrena cineraria*	(Linnaeus, 1758)	x	x
Andrena dorsata*	(Kirby, 1802)	x	x
*Andrena falsifica*	Perkins, 1915	x	
*Andrena flavipes*	Panzer, 1799	x	x
*Andrena fulva*	(Müller, 1766)	x	x
*Andrena fulvata*	Stöckhert, 1930		x
*Andrena fulvida*	Schenck, 1853	x	
*Andrena gravida*	Imhoff, 1832	x	x
*Andrena haemorrhoa*	(Fabricius, 1781)	x	x
*Andrena hattorfiana*	(Fabricius, 1775)	x	
*Andrena helvola*	(Linnaeus, 1758)		x
*Andrena humilis*	Imhoff, 1832	x	x
*Andrena intermedia*	Thomson, 1870	x	
*Andrena labialis*	(Kirby, 1802)		x
*Andrena labiata*	Fabricius, 1781	x	x
*Andrena lagopus*	Latreille, 1809		x
*Andrena lathyri*	Alfken, 1899	x	
*Andrena minutula*	(Kirby, 1802)		x
*Andrena minutuloides*	Perkins, 1914	x	x
*Andrena nigroaenea*	(Kirby, 1802)	x	x
Andrena nigroolivacea*	Dours, 1873		x
*Andrena nitida*	(Müller, 1776)	x	x
*Andrena ovatula* complex*	(Kirby, 1802)	x	x
*Andrena pandellei*	Pérez, 1895	x	x
*Andrena praecox*	(Scopoli, 1763)	x	x
*Andrena schencki*	Morawitz, 1866		x
*Andrena scotica*	Perkins, 1916	x	x
*Andrena strohmella*	Stöckhert, 1928		x
*Andrena subopaca*	Nylander, 1848	x	x
Andrena tibialis*	(Kirby, 1802)	x	x
*Andrena vaga*	Panzer, 1799	x	x
*Andrena ventralis*	Imhoff, 1832	x	
Andrena wilkella*	(Kirby, 1802)	x	
*Anthidium punctatum*	Latreille, 1809	x	
*Anthophora aestivalis*	(Panzer, 1801)	x	x
*Apis mellifera*	Linnaeus, 1758	x	x
*Bombus argillaceus*	Scopoli, 1763	x	
*Bombus barbutellus*	(Kirby, 1802)		x
*Bombus bohemicus*	Seidl, 1838	x	x
*Bombus campestris*	(Panzer, 1801)	x	
*Bombus cryptarum*	(Fabricius, 1775)	x	
Bombus hortorum*	(Linnaeus, 1761)	x	x
*Bombus humilis*	Illiger, 1806	x	x
*Bombus hypnorum*	(Linnaeus, 1758)		x
*Bombus jonellus*	(Kirby, 1802)	x	
Bombus lapidarius*	(Linnaeus, 1758)	x	x
Bombus lucorum*	(Linnaeus, 1761)	x	x
*Bombus pascuorum*	(Scopoli, 1763)	x	x
*Bombus pratorum*	(Linnaeus, 1761)	x	x
Bombus ruderarius*	(Müller, 1776)	x	x
*Bombus* sp.			x
*Bombus soroeensis*	(Fabricius, 1777)	x	
*Bombus rupestris*	(Fabricius, 1793)		x
*Bombus sylvarum*	(Linnaeus, 1761)	x	x
Bombus terrestris*	(Linnaeus, 1758)	x	x
*Bombus vestalis*	(Geoffroy, 1785)		x
*Ceratina cyanea*	(Kirby, 1802)	x	
*Chelostoma distinctum*	(Stoeckhert, 1929)	x	
*Chelostoma florisomne*	(Linnaeus, 1758)	x	x
*Coelioxys conoideus*	(Illiger, 1806)	x	
*Coelioxys mandibularis*	Nylander, 1848	x	
*Colletes cunicularius*	(Linnaeus, 1761)	x	x
*Dasypoda hirtipes*	(Fabricius, 1793)		x
*Eucera longicornis*	(Linnaeus, 1758)	x	
*Eucera nigrescens*	Pérez, 1879	x	x
*Halictus langobardicus*	Blüthgen, 1944		x
*Halictus maculatus*	Smith, 1848	x	x
*Halictus scabiosae*	(Rossi, 1790)	x	x
*Halictus simplex* complex	Blüthgen, 1923	x	x
*Heriades truncorum*	(Linnaeus, 1758)	x	x
*Hoplitis adunca*	(Panzer, 1798)	x	x
*Hoplitis leucomelana*	(Kirby, 1802)	x	x
*Hoplitis mitis*	(Nylander, 1852)	x	
*Hoplitis tridentata*	(Dufour & Perris, 1840)		x
*Hylaeus angustatus*	(Schenck, 1861)	x	x
*Hylaeus brevicornis*	Nylander, 1852	x	x
*Hylaeus communis*	Nylander, 1852	x	
*Hylaeus confusus*	Nylander, 1852	x	
*Hylaeus cornutus*	Curtis, 1831		x
*Hylaeus difformis*	(Eversmann, 1852)		x
*Hylaeus dilatatus*	(Kirby, 1802)		x
*Hylaeus gredleri*	Förster, 1871	x	
*Hylaeus hyalinatus*	Smith, 1842	x	x
*Hylaeus nigritus*	(Fabricius, 1798)	x	x
*Hylaeus paulus*	Bridwell, 1919	x	
*Hylaeus signatus*	(Panzer, 1798)		x
*Hylaeus sinuatus*	(Schenck, 1853)	x	
*Hylaeus styriacus*	Förster, 1871	x	x
*Lasioglossum aeratum*	(Kirby, 1802)	x	
*Lasioglossum albipes*	(Fabricius, 1781)	x	
Lasioglossum angusticeps*	(Perkins, 1895)		x
*Lasioglossum calceatum*	(Scopoli, 1763)	x	x
*Lasioglossum convexiusculum*	(Schenck, 1853)	x	
*Lasioglossum fulvicorne*	(Kirby, 1802)	x	
*Lasioglossum glabriusculum*	(Morawitz, 1872)		x
*Lasioglossum interruptum*	(Panzer, 1798)	x	x
*Lasioglossum laticeps*	(Schenck, 1868)	x	x
*Lasioglossum lativentre*	(Schenck, 1853)	x	x
*Lasioglossum leucopus*	(Kirby, 1802)	x	
*Lasioglossum leucozonium*	(Schrank, 1781)	x	x
*Lasioglossum lucidulum*	(Schenck, 1861)	x	
*Lasioglossum malachurum*	(Kirby, 1802)		x
*Lasioglossum minutissimum*	(Kirby, 1802)		x
Lasioglossum morio*	(Fabricius, 1793)	x	x
*Lasioglossum nigripes*	(Lepeletier, 1841)	x	
*Lasioglossum nitidulum*	(Fabricius, 1804)	x	x
*Lasioglossum pauperatum*	(Brullé, 1832)		x
*Lasioglossum pauxillum*	(Schenck, 1853)	x	x
*Lasioglossum politum*	(Schenck, 1853)	x	
*Lasioglossum punctatissimum*	(Schenck, 1853)	x	x
*Lasioglossum puncticolle*	(Morawitz, 1872)		x
*Lasioglossum sexnotatum*	(Kirby, 1802)	x	
*Lasioglossum villosulum*	(Kirby, 1802)	x	x
*Lasioglossum xanthopus*	(Kirby, 1802)		x
*Lasioglossum zonulum*	(Smith, 1848)	x	x
*Megachile centuncularis*	(Linnaeus, 1758)	x	
*Megachile circumcincta*	(Kirby, 1802)	x	x
*Megachile ligniseca*	(Kirby, 1802)	x	
*Megachile versicolor*	Smith, 1844	x	
*Megachile willughbiella*	(Kirby, 1802)		x
*Nomada armata*	Herrich‐Schäffer, 1839	x	
*Nomada fabriciana*	(Linnaeus, 1767)		x
*Nomada ferruginata*	(Linnaeus, 1767)	x	
*Nomada flava*	Panzer, 1798		x
*Nomada flavoguttata*	(Kirby, 1802)		x
*Nomada fucata*	Panzer, 1798		x
*Nomada fulvicornis*	Fabricius, 1793	x	
*Nomada goodeniana*	(Kirby, 1802)		x
*Nomada lathburiana*	(Kirby, 1802)	x	x
*Nomada leucophthalma*	(Kirby, 1802)	x	
*Nomada ruficornis*	(Linnaeus, 1758)		x
*Nomada sexfasciata*	(Panzer, 1799)	x	
*Osmia aurulenta*	Panzer, 1799	x	
*Osmia bicolor*	(Schrank, 1781)	x	x
*Osmia bicornis*	(Linnaeus, 1758)	x	x
*Osmia caerulescens*	(Linnaeus, 1758)	x	x
*Osmia labialis*	Pérez, 1879	x	
*Osmia leaiana*	(Kirby, 1802)		x
*Osmia xanthomelana*	Kirby, 1802	x	
*Panurgus calcaratus*	(Scopoli, 1763)	x	
*Seladonia confusa*	(Smith, 1853)	x	
Seladonia leucahenea*	(Ebmer, 1972)		x
*Seladonia subaurata*	(Rossi, 1792)	x	x
*Seladonia tumulorum*	(Linnaeus, 1758)	x	x
*Sphecodes albilabris*	(Fabricius, 1793)	x	
*Sphecodes crassus*	Thomson, 1870	x	x
*Sphecodes ephippius*	(Linnaeus, 1767)	x	x
*Sphecodes ferruginatus*	Hagens, 1882	x	
*Sphecodes geoffrellus*	(Kirby, 1802)	x	
*Sphecodes monilicornis*	(Kirby, 1802)		x
*Sphecodes niger*	Hagens, 1874	x	
*Sphecodes puncticeps*	Thomson, 1870	x	x
*Trachusa byssina*	(Panzer, 1798)	x	

A maximum of 10 individuals per species and sex/caste (depending on the availability) were air‐dried and needled to measure their intertegular distances (ITD). The ITD of bees correlates with body size (Kendall et al. 2019) and was used as a proxy of biomass (Cane 1987). We measured the ITD using a macroscope with apochromatic zoom system and digital calliper (Leica Z6Apo). Finally, we calculated average ITD estimates and inferred biomasses for each species following Cane (1987).

Ecological traits were assigned to species based on Westrich (2018) consisting of the following variables: pollen selection (polylectic, oligolectic), nesting biology (below‐ground nesting, above‐ground nesting) and sociality (social, solitary, parasite). Species lacking corroborated knowledge for a trait, as well as species for which more than one state of a trait is known, were not used under the corresponding trait. This dataset allowed us to better understand species assemblages and the combinations of functional traits of bees in different environments.

### 2.3 Quantification of Flower Colours

In all eight sampling periods, we assessed white, yellow and blue flower colours of all study sites through drone flights providing aerial images of the immediate environmental context of the pan traps during sunny and calm weather conditions between 09:00 AM and 03:30 PM on the same day the traps were emptied. Floral colours were comparable with pan trap colours, which are commonly used to collect wild bees (see for example Westerberg et al. 2021). Drone images have the advantage over orthophotos that they have fewer limitations in the visual detection of small‐scale structures and vegetation cover (Zimmermann et al. 2023). Mini drones of the type DJI Mini 2 took pictures from 10 to 50 m vertical distance, orthogonal to the mid pan trap. However, we only used pictures from 10 m flying height for the final data analyses since the pictures taken from greater heights were too low in resolution. At 10 m drone flight height, images covered 385 m^2^ of the immediate surrounding of the pan traps. Using the python script ‘ColorDetect’, written in Anaconda Navigator 2.1.1 (Anon 2020) and publicly available (https://github.com/chrishaider/FlowerColorDetect), we filtered all 224 drone images (2 regions × 2 habitat types × 7 study sites × 8 sampling periods) by the three flower colours. Briefly, for white and yellow, the white and yellow pan traps of each image were used to identify the hue‐saturation‐value (hsv) ranges, and for blue, a fixed hsv range from (90,100,0) to (145,255,255) was used; upon scrutiny by eye of the thereby included and excluded parts of the image, that is, checking for false positives and false negatives, these colour thresholds were adjusted when necessary; this procedure was ground validated in a pilot (not shown). Then, for each study site, the pixels per colour were expressed as percentage of all pixels of the image (after subtraction of the area covered by the pan traps, that is, 0.039 m^2^ per image) averaged across sampling periods.

### 2.4 Quantification of Habitat Structures

We digitised the immediate environment of the pan traps with ArcGIS Pro (2.7.0) and QGIS (3.27) using orthophotos with a resolution of 20 cm, provided by the Tyrolean government (Land Tirol—data.gv.at) and Luxembourg government (https://www.geoportail.lu) to derive three variables of habitat structure, that is ‘number of polygons’, ‘eco tree’ and ‘eco fields’. In more detail, we drew a circle with a 150 m radius around each of the three pan‐trap sets of each site and merged these three overlapping circles into a single ellipse per site (representing 79,654 m^2^). We then quantified habitat structures as polygons within this ellipse including structures such as boundary ridges, waysides, brook sides, borders of houses, gardens and power poles, as well as single trees, bushes, hedgerows and woods. For the latter four, we quantified the treetop coverage as it can be expected that it has a stronger impact than the trunk on the micro‐environmental scale. The number of polygons within the ellipse of each site was counted and further used as the variable ‘number of polygons’. Next, we subtracted the perimeter of the ellipse (as it did not represent the true border of a habitat structure) from the sum of perimeters of all polygons inside the ellipse and then divided this value by 2 to prevent that polygon perimeters were double counted (any bordering line between two polygons was quantified for both of them initially), thus obtaining the ecotone length of all polygons. Of this length, the ecotone lengths between, on the one hand, single trees, bushes, hedgerows and woods and, on the other hand, all other polygons were obtained and further used as the variable ‘eco tree’. By subtracting the value of ‘eco tree’ from the ecotone length of all polygons the variable ‘eco fields’ was obtained, thus representing the ecotone lengths of fields without trees or bushes.

### 2.5 Statistical Analysis

All analyses were conducted in R 3.6.1 (R Core Team 2021), except when noted otherwise, separately for the two regions, AT and LU. For each of the 14 study sites, we summed up the results from all eight sampling periods, resulting in a single set of data per study site. To check for potential spatial autocorrelation, the abundance data of wild bee species were used to calculate Euclidean distances among all 14 sites per region, and a correlation analysis was run of the Euclidean distances against the geographic distances among sites for AT and LU separately; for this, PAST v 4.17 was used (Hammer et al. 2001). Analysing together the 14 sites per region, we then fitted simple linear regressions each for wild bee abundance, species richness and biomass to the habitat‐heterogeneity proxies (number of polygons, eco tree and eco fields) separately, correcting for multiple comparison within the three analyses of the same wild bee data via Bonferroni *p* value adjustment using PAST v 4.17. The package ‘RADanalysis’ (Saeedghalati et al. 2016) was used to calculate and visualise a rank abundance distribution analysis (RADanalysis), providing insights into the relative abundance of species within wild bee communities documented on the two habitat types. We applied the packages ‘tidyverse’ and ‘ggvenn’ to produce boxplots and Venn diagrams (Wickham et al. 2019; Gao et al. 2021). We calculated the Shannon and evenness indices for both habitat types in each region (EF_AT, IF_AT; EF_LU, IF_LU) using the function ‘diversity’ implemented in the ‘vegan’ package (Oksanen et al. 2017). We compared characteristics of species diversity (wild bee abundance, species richness, Shannon index, evenness, biomass) and ecological traits (pollen selection, nesting behaviour, sociality) among habitat types per region by testing for differences with a Wilcoxon Rank Sum Test using the function ‘wilcox.test’. Significant *p*‐values of ≦ 0.05 were added to the respective plots.

We evaluated species richness and sample coverage using the package ‘iNEXT’ (Hsieh et al. 2022). Species richness per habitat type was represented as a function of sampling units with a 95% confidence interval obtained through bootstrapping. We extracted the rarefied estimates for sample coverage using the function ‘estimateD’.

Finally, non‐metric multidimensional scaling (NMDS) plots based on Bray–Curtis dissimilarity (function ‘vegdist’, method = ‘bray’) were used to illustrate community compositions, together with the results of the analysis of similarity (ANOSIM) using the package ‘vegan’ (Oksanen et al. 2017). In doing so, we tested again for differences between habitat types within regions (EF_AT vs. IF_AT; EF_LU vs. IF_LU) applying the vector‐fitting function ‘envfit’ with 9999 permutations for environmental structures (eco tree, eco fields, number of polygons) and abundant flower colours (white, yellow, blue). Derived *p*‐values for environmental factors were adjusted by applying the Bonferroni correction using the function ‘p.adjust’.

## 3 Results

### 3.1 Wild Bee Data Collected

From AT, we collected 1988 individuals of 117 wild bee species in 21 genera in EF_AT and IF_AT (Table 1, Table S3). The top three most species‐rich genera were *Andrena* with 27 species (15.0% of all specimens), *Lasioglossum* with 20 species (58.8%) and *Bombus* with 15 species (9.6%). Insufficient data recording once occurred when pan traps were removed at site EF_AT Plot 5 during the first collection period in mid‐April. The seasonal distribution of honeybee abundance on the different plots is depicted in Figure S3.

From LU, we collected 3287 wild bees of 105 wild bee species in 17 genera in EF_LU and IF_LU (Table 1, Table S4). The top three genera for Luxembourg were the same as for AT, but in a different order: *Andrena* was represented by 30 species (40.2% of all specimens), *Lasioglossum* by 18 species (42.3%) and *Bombus* by 15 species (7.2%). Out of these 105 species, five represented first records for the country ( *A. nigroolivacea* , *Hylaeus styriacus* Förster, 1871, *L. angusticeps* , *Lasioglossum pauperatum* (Brullé, 1832) and *S. leucahenea*). Together with other wild bee first records made after the initiation of this study (i.e., Cantú‐Salazar et al. 2021; Siebenaler et al. 2022) our findings of *A. nigroolivacea* , *L. angusticeps* , *L. pauperatum* and *S. leucahenea* were already considered in a European meta‐analysis for wild bees (Reverté et al. 2023). Insufficient data recording occurred twice, when (i) three pan traps were destroyed (IF_LU Plot 6 between 9.5.2021 and 29.5.2021) or (ii) washed out due to heavy rainfall (EF_LU Plot 5 between 3.7.2021 and 17.7.2021). Trapping in LU was completely halted between 14.6.2021 and 25.06.2021 due to potential mowing activities, which, however, did not happen due to wet weather conditions. The seasonal distribution of honeybee abundance on the different plots is depicted in Figure S3.

Neither in AT nor in LU, the correlation of Euclidean distances of wild bee communities with geographic distances for the 14 study sites per region was significant (AT: *r* = 0.040, *p* = 0.709; LU: *r* = 0.065, *p* = 0.543). This indicated a lack of spatial autocorrelation in the data for both regions.

Social *Lasioglossum* species counted for the highest number of collected individuals per species in both habitat types of both regions (Figure 3). Seventy‐six species were collected with a maximum of five individuals, and 36 species were represented by just a single individual. Eudominant species with more than 200 individuals per species were *Lasioglossum morio* (Fabricius, 1793) and *Lasioglossum nigripes* (Lepeletier, 1841) in EF_AT, *Lasioglossum malachurum* (Kirby, 1802) and *Andrena cineraria* in EF_LU, and *L. malachurum* and *Andrena flavipes* Panzer, 1799 in IF_LU. RAD analysis showed that EF_LU and IF_LU in LU host a similar composition of eudominant species as two of the top five abundant species ( *L. malachurum* , *A. cineraria* ) were collected in both habitat types. This contrasts with habitat types in AT, where the most abundant species differed between habitat types. Besides the decreased species abundance curve, the intensively farmed habitat type IF_AT displayed a higher species evenness (Figure S4) and individual numbers for all species below the threshold of 200.

**FIGURE 3:**
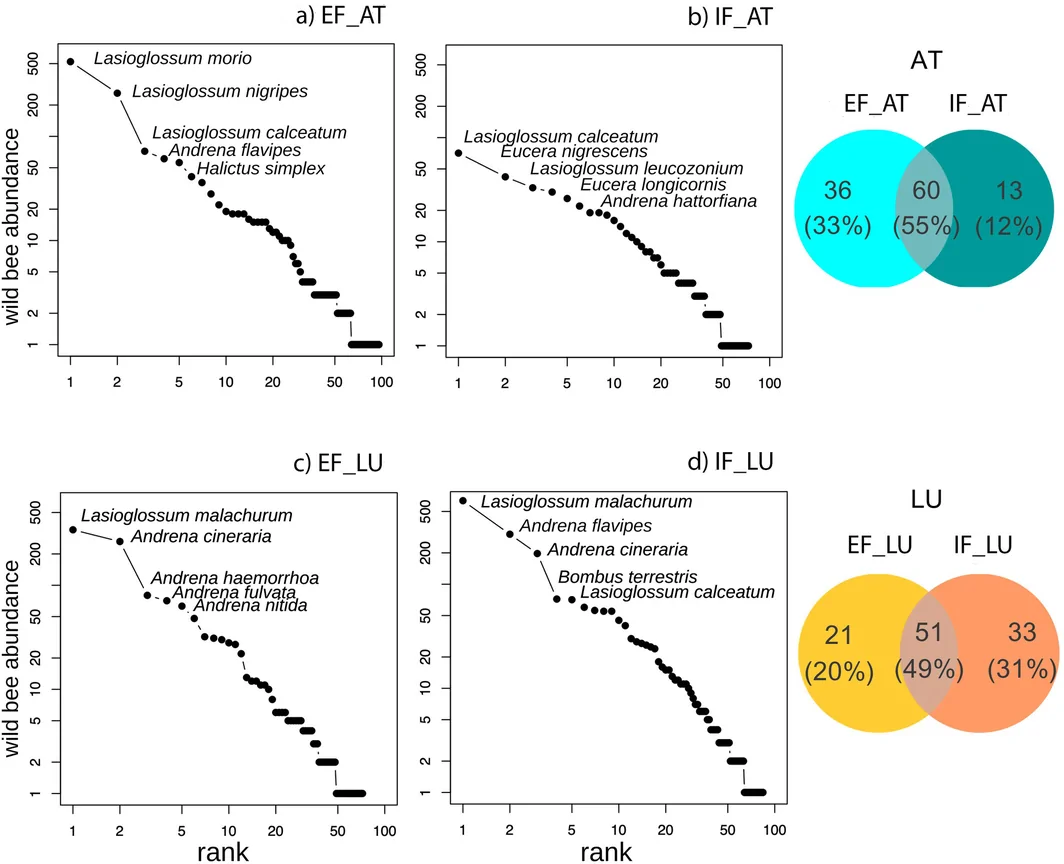
Rank abundance analysis for the two investigated habitat types in Austria (AT) and Luxembourg (LU) (EF_AT and EF_LU as extensively used grassland; IF_AT and IF_LU as formerlyintensively farmed land) show that (eu)dominant species were social, below‐ground nesting wild bees (e.g., *Lasioglossum morio* , *Lasioglossum nigripes, Lasioglossum calceatum, Lasioglossum leuconium*) or species establishing aggregates (e.g., *Andrena cineraria, Andrena flavipes*). The Venn diagrams on the right indicate that EF_AT habitats in Austria host more species exclusively than IF_AT, and that IF_LU habitats in Luxembourg host more species exclusively than EF_LU.

In both regions, the intersections of species found in both habitat types were similar in numbers (Venn diagrams in Figure 3), whereas EF_AT and IF_LU represented a larger number of unique wild bee species compared with IF_AT and EF_LU.

### 3.2 Wild Bee Diversity and Habitat Heterogeneity

In At, the number of polygons per site ranged from 32 to 145 (75.6 arithmetic mean ± 36.0 standard deviation, SD; details in Table 2), the length of ecotones involving single trees, bushes, hedgerows or woods (eco tree) from 31.5 to 3376.7 m (1412.0 ± 1173.0 m), and the length of all other ecotones (eco fields) from 1593.4 to 5409.0 m (3462.4 ± 1155.4 m); in LU, the number of polygons per site ranged from 9 to 60 (30.4 ± 18.7), eco tree from 0.0 to 2202.0 m (914.4 ± 688.1 m), and eco fields from 1448.5 to 4407.9 m (2293.8 ± 942.4 m). To assess a potential influence of habitat heterogeneity on wild bees, we analysed together the 14 sites per region, using the number of polygons and ecotone lengths (eco tree, eco fields). Among all regressions of wild bee abundance, species richness, and biomass, only abundance against eco tree in AT was significant (adjusted *p* = 0.043, *R* ^2^ = 0.406), with a positive relationship.

**TABLE 2:** Wild bee abundance, species richness and biomass (based on the average intertegular distance of max. 10 individuals per sex and species) as well as Shannon index and evenness (both dimensionless) for the investigated habitat types in Austria (AT) and Luxembourg (LU), extensively used grassland (EF) and ecologically improved formerly intensively farmed land (IF)the heterogeneity of each site quantified via the number of polygons (polygon) and lengths of ecotones involving single trees, bushes, hedgerows or woods (eco tree) and of ecotones not involving these (eco fields), as well as flower colours (white, yellow, blue; % of pixels of all pixels of a site's image, averaged across the eight sampling periods) quantified using drone photography.

Country	Habitat	Sites	Abundance (*n* individuals)	Species richness (*n* species)	Biomass (mg)	Shannon index	Evenness	Polygons (*n* elements)	Eco tree (m)	Eco fields (m)	White (%)	Yellow (%)	Blue (%)
AT	EF_AT	RS‐EF1	97	28	1362.8	2.79	0.84	122	2774.7	3733.3	0.0036	0.0865	0.0020
AT	EF_AT	RS‐EF2	168	38	1668.2	2.34	0.64	59	1520.5	1882.3	0.0001	0.0071	0.0003
AT	EF_AT	RS‐EF3	242	32	1949.3	1.73	0.50	72	2884.6	1593.4	0.0439	0.0944	0.0155
AT	EF_AT	RS‐EF4	209	40	1799.5	2.45	0.66	37	2011.9	2340.1	0.0178	0.1012	0.0223
AT	EF_AT	RS‐EF5	250	38	4192.1	2.75	0.76	107	691.5	5409.0	0.0140	0.0231	0.0268
AT	EF_AT	RS‐EF6	152	36	2054.9	2.68	0.75	115	2918.2	3223.9	0.0596	0.0325	0.0056
AT	EF_AT	RS‐EF7	366	36	4002.3	1.62	0.45	145	3376.6	4210.5	0.0011	0.0186	0.0000
AT	IF_AT	PS‐IF8	71	26	1075.1	2.91	0.89	99	898.0	4565.5	0.0457	0.0643	0.0043
AT	IF_AT	PS‐IF9	38	18	801.9	2.60	0.90	53	789.3	2763.8	0.0997	0.2083	0.0045
AT	IF_AT	PS‐IF10	52	23	1453.9	2.75	0.88	55	43.5	4740.7	0.0666	0.0783	0.0038
AT	IF_AT	PS‐IF11	65	27	1880.0	2.83	0.86	74	874.9	4662.7	0.0579	0.1075	0.0105
AT	IF_AT	PS‐IF12	135	36	3836.2	2.83	0.79	32	31.5	2914.6	0.0575	0.1400	0.0124
AT	IF_AT	PS‐IF13	48	22	1634.6	2.74	0.89	34	44.9	3678.4	0.0109	0.0351	0.0024
AT	IF_AT	PS‐IF14	95	35	2626.3	3.21	0.90	55	907.8	2755.5	0.1610	0.1290	0.0069
LU	EF_LU	BW1	152	28	2746.5	2.34	0.70	14	1627.6	1809.4	0.0471	0.9532	0.3787
LU	EF_LU	BW2	280	43	5177.8	2.74	0.73	9	313.3	1666.8	0.1520	3.0893	0.0305
LU	EF_LU	BW3	88	20	1626.1	2.55	0.85	58	2202.0	3991.1	0.0144	0.2245	0.2357
LU	EF_LU	BW4	224	29	7304.2	2.61	0.78	25	1288.6	1559.5	0.1365	2.6904	0.0261
LU	EF_LU	BW5	184	28	3228.9	2.12	0.64	22	941.6	2236.3	0.1897	0.6572	0.0651
LU	EF_LU	BW6	158	28	3284.3	2.59	0.78	47	1243.7	1802.9	0.4823	0.3065	0.0161
LU	EF_LU	BW7	156	28	3909.3	2.32	0.70	30	389.9	3098.8	0.6420	0.1317	0.0050
LU	IF_LU	BU1	390	33	6875.7	1.99	0.57	14	0.0	1699.3	0.2177	0.0453	1.5232
LU	IF_LU	BU2	273	40	3813.4	2.64	0.72	10	0.0	1448.5	0.0687	0.0126	0.0413
LU	IF_LU	BU3	285	44	3963.6	2.53	0.67	46	426.4	2879.7	0.0242	0.0234	0.0000
LU	IF_LU	BU4	238	35	6469.2	2.98	0.84	21	843.5	1761.9	0.0664	0.0713	1.2227
LU	IF_LU	BU5	314	48	5434.1	2.90	0.75	15	691.7	1662.0	0.0378	0.0274	1.3038
LU	IF_LU	BU6	413	39	5698.2	2.28	0.62	54	1979.1	4407.9	0.0138	0.1162	0.2277
LU	IF_LU	BU7	132	36	2050.5	3.02	0.84	60	853.9	2088.5	0.0994	0.0082	0.0003

### 3.3 Wild Bee Diversity Per Habitat Type

Wild bee abundance was significantly higher in EF_AT than IF_AT (EF_AT: 1484 individuals; arithmetic mean 212.00 ± 86.44 SD per site; IF_AT: 504 individuals; 72.00 ± 33.37; *p* = 0.003) and significantly lower in EF_LU than IF_LU (EF_LU: 1242 individuals, 177.43 ± 60.85; IF_LU: 2045 individuals, 292.14 ± 94.56; *p* = 0.041; Figure 4; Table 2). Species richness was significantly higher in EF_AT than IF_AT (EF_AT mean: 35.57 ± 4.32; IF_AT 26.71 ± 6.68; *p* = 0.015) with a total of 96 species equalling 56.1% of total biomass and 73 species equalling 43.9% of biomass, respectively. Species richness was significantly lower in EF_LU than IF_LU (EF_LU mean: 29.14 ± 6.84; IF_LU 39.29 ± 5.28; *p* = 0.014; Figure 4) with 72 species in EF_LU equalling 45.3% of biomass and 84 species in IF_LU equalling 55.7% of total biomass. These results were supported by a rarefaction‐extrapolation analysis for species richness and number of collected individuals per habitat type representing similar rarefied estimates of sample coverage (EF_AT: 0.97 and IF_AT: 0.98; EF_LU: 0.99 and IF_LU: 0.98; Figure 5) indicating a similar sampling effort for both habitat types in both regions. As a function of species richness and abundance, we calculated the Shannon and evenness indices with significant differences between habitat types only in AT (EF_AT and IF_AT: Shannon Index *p* = 0.022; Evenness *p* = 0.003; Figure S4).

**FIGURE 4:**
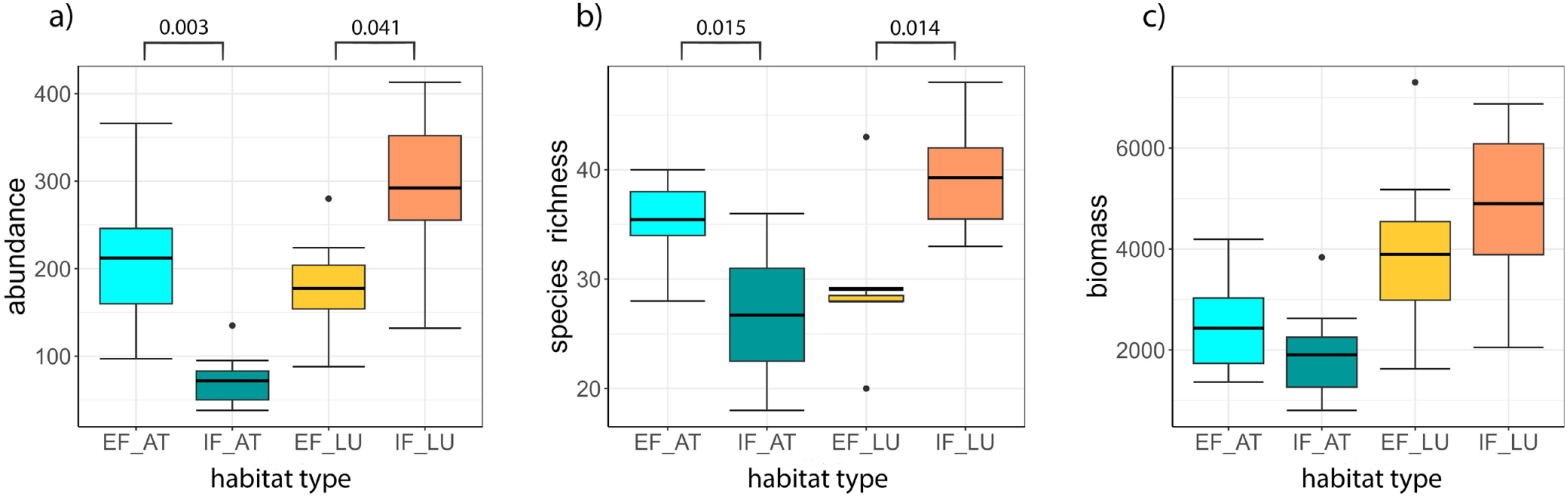
Differences and opposite trends in wild bee (a) abundance, (b) species richness, and (c) biomass per habitat type (Austria: EF_AT = extensively used grassland vs. IF_AT = ecologically improved formerly intensively farmed land; Luxembourg: EF_LU = extensively used grassland vs. IF_LU = ecologically improved formerly intensively farmed land). For wild bee abundance and species richness, the total number of individuals per habitat and total number of species were counted. Biomass was derived as a function of the intertegular distance and averaged from max. 10 individuals. The *p*‐values indicating a significant difference between habitats per region were calculated with a Wilcoxon rank sum test.

**FIGURE 5:**
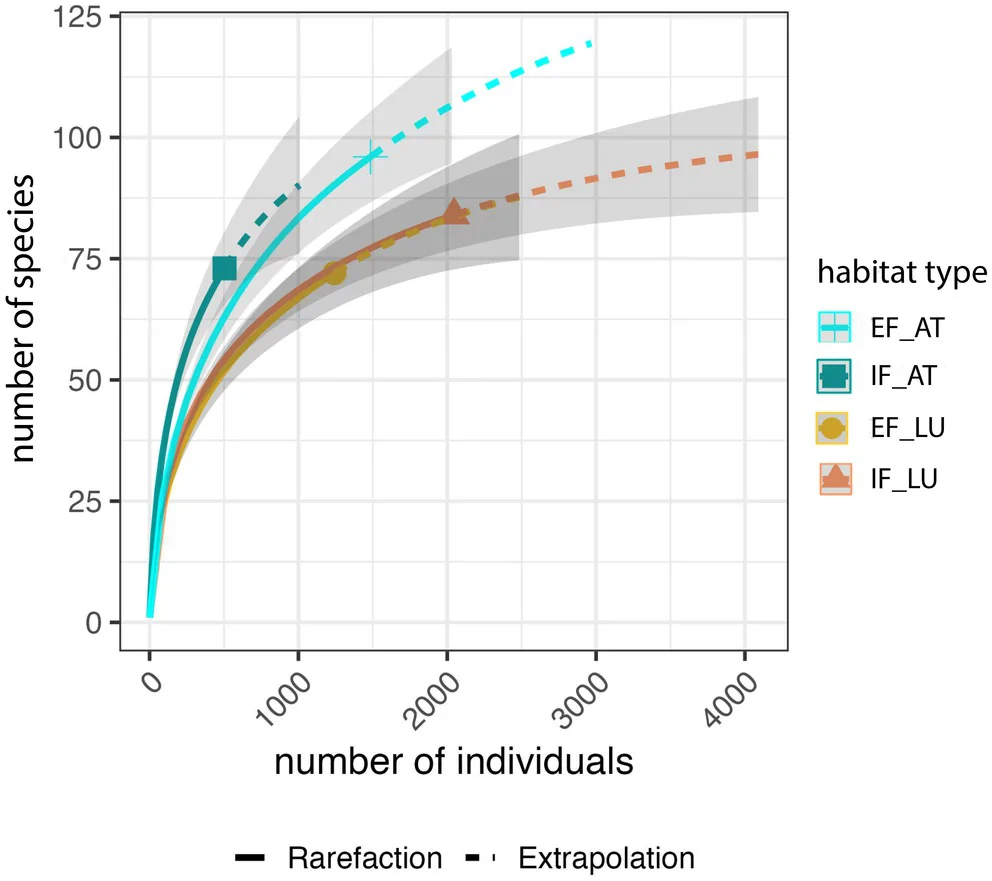
Rarefaction‐extrapolation shows for both investigated habitat types in both regions a similar sampling effort and no saturation. The curves of the habitat types in Luxembourg (EF_LU = extensively used grassland, IF_LU = ecologically improved formerly intensively farmed land) were less steep than those in Austria (EF_AT = extensively used grassland, IF_AT = ecologically improved formerly intensively farmed land).

Biomass did not differ significantly between EF_AT and IF_AT (EF_AT 17,028.97 mg; mean 2432.71 ± 1159.43 SD per site; IF_AT 13,307.91 mg; mean: 1901.13 ± 1035.42; *p* = 0.250; Table 2, Figure 4) nor between EF_LU and IF_LU (EF_LU 27,277.24 mg; mean: 3896.75 ± 1850.77; IF_LU 34,304.63 mg; mean: 4900.66 ± 1706.13; *p* = 0.011).

### 3.4 Species Composition of Communities Based on Ecological Traits

The habitat types EF and IF mostly differed in the species compositions of their regional communities when considering individual ecological traits of species separately (nesting biology, pollen selection, sociality) (Figure S5, Table S5). Thus, of 73 below‐ground nesting (or endogaeic) species in AT, 18 occurred on exclusively EF_AT sites and 11 on exclusively IF_AT sites; however, species richness did not differ between the two habitat types (EF_AT arithmetic mean 25.57 ± 3.95 SD; IF_AT 22.14 ± 5.15). In LU, nine of 66 below‐ground nesting species occurred on exclusively EF_LU sites and 19 on exclusively IF_LU sites; here, species richness was lower in EF_LU than IF_LU (EF_LU 23.29 ± 5.25; IF_LU 30.57 ± 5.03; *p* = 0.029). Of 35 above‐ground nesting (or epigaeic) species in AT, 17 occurred on exclusively EF_AT sites and five on exclusively IF_AT sites; species richness was higher in EF_AT than IF_AT (EF_AT 10.57 ± 3.10; IF_AT 5.86 ± 2.12; *p* = 0.010). In LU, of 28 above‐ground nesting species, six occurred on exclusively EF_LU sites, 11 on exclusively IF_LU sites; species richness was lower in EF_LU than IF_LU (EF_LU 5.86 ± 1.86; IF_LU 9.29 ± 3.25; *p* = 0.050). Of 21 oligolectic species in AT, six occurred on exclusively EF_AT sites and eight on exclusively IF_AT sites; species richness did not differ between habitat types (EF_AT 4.14 ± 1.25 SD; IF_AT 4.71 ± 1.70). In LU, of 16 oligolectic species, five occurred on exclusively EF_LU sites and six on exclusively IF_LU sites; species richness did not differ between habitat types (EF_LU 2.29 ± 1.80; IF_LU 3.43 ± 1.72). Of 79 polylectic species in AT, 27 occurred on exclusively EF_AT sites and nine on exclusively IF_AT sites; species richness was higher in EF_AT than IF_AT (EF_AT 28.71 ± 2.87; IF_AT 20.43 ± 4.50; *p* = 0.007). In LU, of 74 polylectic species, 10 occurred on exclusively EF_LU sites and 24 on exclusively IF_LU sites; species richness was lower in EF_LU than IF_LU (EF_LU 24.14 ± 4.10; IF_LU 33.71 ± 5.41; *p* = 0.006). Of 22 social species in AT, four occurred on exclusively EF_AT sites and one on exclusively IF_AT sites; species richness did not differ between EF_AT and IF_AT (EF_AT 11.29 ± 1.60 SD; IF_AT 9.43 ± 2.99). In LU, of 20 social species, one occurred on exclusively EF_LU sites and five on exclusively IF_LU sites; species richness was lower in EF_LU and IF_LU (EF_LU 8.86 ± 1.77; IF_LU 12.57 ± 3.41; *p* = 0.029). Of 73 solitary species in AT, 28 occurred on exclusively EF_AT sites and 15 on exclusively IF_AT sites; species richness was higher in EF_AT than IF_AT (EF_AT 19.14 ± 2.67; IF_AT 14.57 ± 3.05; *p* = 0.020). In LU, of 70 solitary species, 14 occurred on exclusively EF_LU sites and 25 on exclusively IF_LU sites; species richness was lower in EF_LU and IF_LU (EF_LU 17.57 ± 4.65; IF_LU 24.57 ± 3.87; *p* = 0.015) in LU. Of 17 parasitic species in AT, 10 occurred on exclusively EF_AT sites and five on exclusively IF_AT sites; species richness did not differ between habitat types (EF_AT 2.43 ± 1.72; IF_AT 1.71 ± 1.60). In LU, of 15 parasitic species, six occurred on exclusively EF_LU sites, three on exclusively IF_LU sites; species richness did not differ between habitat types (EF_LU 2.71 ± 2.21; IF_LU 2.14 ± 2.04).

### 3.5 Wild Bee Communities and Environmental Variables

In AT, the percentage of cover of white flowers per site ranged from 0.01 to 0.16 (0.05 arithmetic mean ± 0.04 SD), that of yellow flowers from 0.01 to 0.21 (0.08 ± 0.06), and that of blue flowers from 0.01 to 0.03 (0.02 ± 0.01); in LU, the corresponding values for white flowers were 0.01 to 0.64 (0.16 ± 0.19), those for yellow flowers 0.01 to 3.09 (0.60 ± 1.01), and those for blue flowers 0.02 to 1.52 (0.38 ± 0.54).

ANOSIM tests for the two regions separately indicated a distinctive difference between habitat types when considering the number of wild bee individuals of all species (Figure 6a) with a significant influence of the length of ecotones involving single trees, bushes, hedgerows or woods (eco tree) in AT (eco tree, *p* < 0.019). This contrasted with analysing community compositions based on their biomass (Figure 6b), as habitat types in AT displayed similar biomasses between EF_AT and IF_AT with greater differences in species composition per habitat type and a significant influence of eco tree (*p* < 0.002). The biomass of wild bee communities in EF_LU and IF_LU habitat types was more different (Figure 6b), but environmental factors were returned as not significant.

**FIGURE 6:**
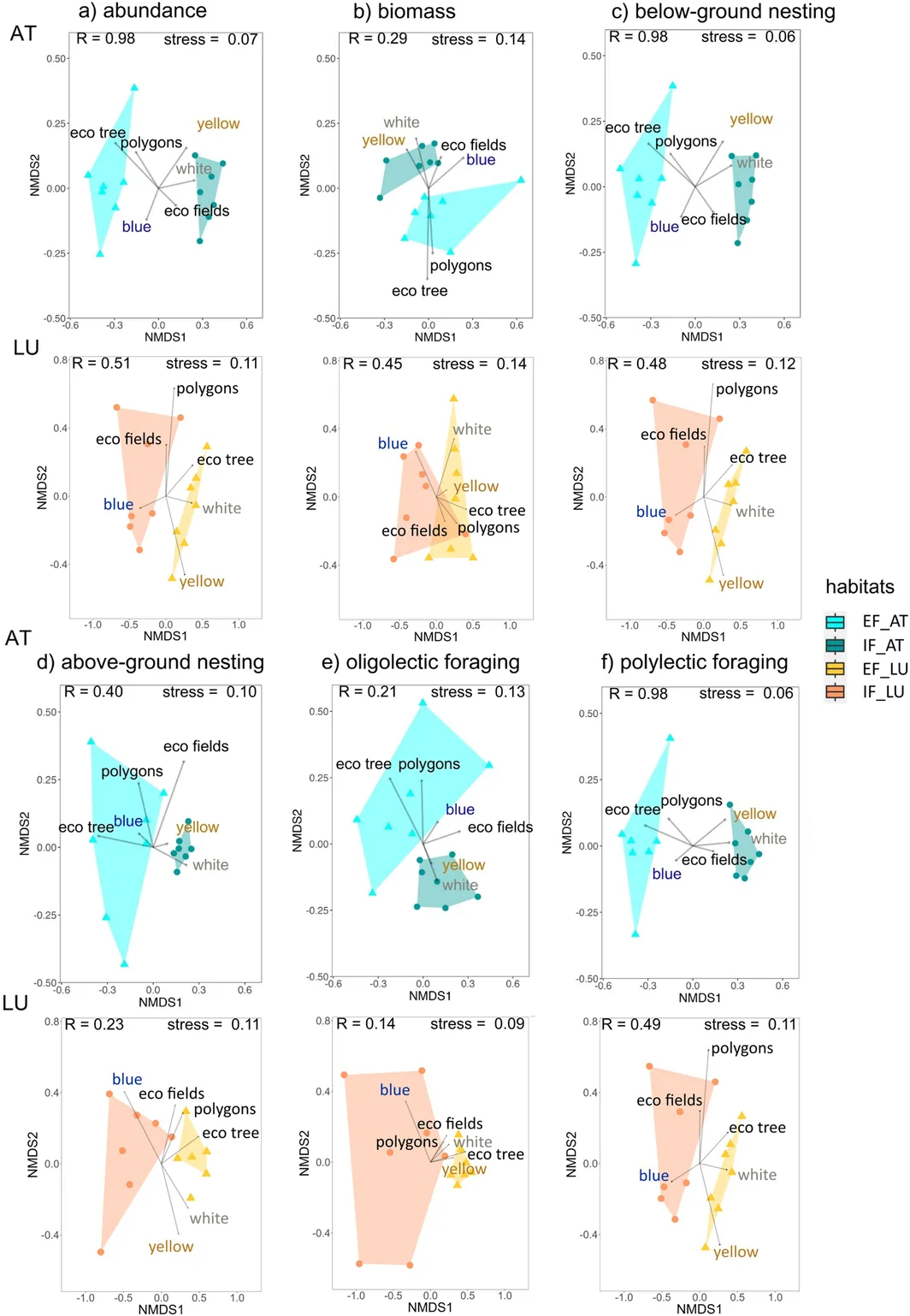
Non‐metric multidimensional scaling (NMDS) plots and *R*‐values derived from analysis of similarity describe the difference between two investigated habitat types in Austria (EF_AT = extensively used grassland, IF_AT = ecologically improved formerly intensively farmed land) in the top plots and two investigated habitat types in Luxembourg (EF_LU = extensively used grassland and IF_LU = ecologically improved formerly intensively farmed land) below. The plots represent community compositions based on (a) abundance values for all species (Austria *n* species = 117, *n* individuals = 1988; Luxembourg *n* species = 105, *n* individuals = 3287); (b) biomass values for all species (Austria total biomass = 30,336.88; Luxembourg total biomass =48,757.17); (c) abundance values for below‐ground nesting species (Austria *n* species = 72, *n* individuals = 1813; Luxembourg *n* species = 66, *n* individuals = 3094); (d) abundance values for above‐ground nesting species (Austria *n* species = 36, *n* individuals = 212; Luxembourg *n* species = 28, *n* individuals = 234); (e) abundance values for oligolectic species (Austria *n* species = 20, *n* individuals = 237; Luxembourg *n* species = 16, *n* individuals = 78); (f) abundance values for polylectic species (Austria *n* species = 80, *n* individuals = 1707; Luxembourg *n* species = 74, *n* individuals = 3153). The vectors for habitat structures and flower colours per habitat indicate which factors drive the differences represented in the ordinations.

When considering separately fractions of the communities according to ecological traits (nesting biology, pollen selection, sociality), additional insights were gained. Thus, species composition including only below‐ground nesting species was different for all habitat types (Figure 6c). Again, eco tree (*p* = 0.023) was tested as a significant factor for EF_AT and IF_AT. This difference was less distinctive in EF_AT and IF_AT including above‐ground nesting species and was not revealed for EF_LU and IF_LU (Figure 6d). The environmental factor with significant influence was the length of ecotones not involving trees or bushes (eco fields, *p* = 0.022) for EF_AT and IF_AT.

Regarding the pollen collection behaviour, we detected a difference in species composition for both regions when considering only polylectic species (Figure 6f) with eco tree (*p* = 0.038) as significant factor between EF_AT and IF_AT. The difference within the communities of oligolectic bees was greater than between habitat types in both regions (Figure 6e). The influencing factor was eco tree (*p* = 0.007) for EF_AT and IF_AT sites.

## 4 Discussion

Central European grassland ecosystems host a rich wild bee diversity. During the 3‐month study period (mid‐April to mid‐July), we collected around 30% of all known species in the respective regions (Austria and Luxembourg). According to our rarefaction analysis, however, this list might be incomplete for both regions. This is in accordance with previous studies that have emphasised the value of grassland for wild bee diversity (Banaszak and Szefer 2014; Ziaja et al. 2018) and is reasonable given that the temporal design of our study did not adequately capture late‐flying, mostly specialist wild bee species.

Our data were obtained by using coloured pan traps (Krahner et al. 2024); yet, these traps are known to exert certain biases: Pan traps might attract bees over distances exceeding the size of the investigated sites, but, in contrast, their capture efficacy depends on the abundance and competing attractiveness of nearby flowers (Westerberg et al. 2021; Mathis et al. 2024). Moreover, pan traps are known to display some bias in sampling bee communities, as for example, large‐bodied bees (e.g., *Bombus* spp. and *Xylocopa* spp.) seem underrepresented as compared with Malaise traps, which in turn underrepresent small bees in their sample (Anderson et al. 2024). In our study, indeed, no *Xylocopa* bee was captured, but pan traps were able to trap, for example, 75% of all known *Bombus* species in Luxembourg. Another bias may have stemmed from our spatial replication of three sets of pan traps per site (i.e., nine traps per site), which was not high; however, in balancing the efforts of the campaign, we rather aimed at achieving the desired number of trap days via an extended sampling period than an extended spatial replication; the generalizability of our findings may be limited by this spatial replication but the numbers of individuals trapped appear generally sufficient for cautious interpretation of our data. In all, given that any such trapping bias should apply to all study sites similarly, we assume that the data collected can be compared across the investigated sites, even if some distortion cannot be ruled out (Mathis et al. 2024; Anderson et al. 2024)—notably the potential underrepresentation of *Bombus* specimens in our samples, which in turn would affect both overall and site‐specific biomass estimates.

We found no indication of spatial autocorrelation in the wild bee community data of either region, and in Section 4.1, we thus validate our initial expectation that (i) heterogeneous habitats host higher wild bee abundance, species richness, and biomass than less heterogeneous ones, in particular in the geographical context of the analysed sites in AT. In Section 4.2, we discuss the more complex pattern revealed for our second expectation that (ii) extensively used grassland hosts a greater species richness and guild diversity of wild bees but lower biomass than ecologically improved formerly intensively farmed land. The expectation was supported for AT, but the situation was different in LU, where the ecologically improved IF_LU sites hosted greater species richness and guild diversity of wild bees, as well as a higher, albeit non‐significantly higher, biomass. In Section 4.3, we attempt making recommendations for the conservation biology of agriculturally dominated land more generally.

### 4.1 Habitat Heterogeneity

For LU, no relation was observed between our habitat heterogeneity proxies and any of the wild bee diversity characteristics analysed (abundance, species richness, biomass; Section 3.2). We assume that existing but not measured micro‐environmental features such as the proportion of open soil patches and the number of unmown vegetation stems played a significant role (L. Hellers, unpublished). The situation awaits future validation when data on more fine‐scaled parameters of habitat heterogeneity than used here will have become available, as it likewise applies to AT.

For AT, and in line with our expectations, we found that with increasing habitat heterogeneity as measured by the length of ecotones involving single trees, bushes, hedgerows or woods (eco tree), wild bee abundance increases. This is in accordance with previous studies highlighting the conservation value of heterogeneous elements in semi‐natural habitats (Twerd and Sobieraj‐Betlińska 2020; Sobieraj‐Betlińska et al. 2023). In more detail, forest edges are microhabitats with often specialised vegetation and open soil patches offering nesting opportunities for endogaeic species and their cuckoo bees (Banaszak and Szefer 2014; Sobieraj‐Betlińska et al. 2023). In addition, above‐ground nesting species benefit from heterogeneous landscapes due to a higher degree of connectivity of habitats (Williams et al. 2010). Also, trees and shrubs, besides herbaceous plants, provide additional food resources, which are usually found in ecotones between forests and fields (Zurbuchen, Cheesman, et al. 2010).

The relevance of habitat heterogeneity to bee communities was also indicated by the results of our species community analyses for AT (Section 3.5)—the significant factors accountable for the differences in species communities were wood ecotones and edges between fields. The lack of additional relations between our habitat heterogeneity proxies and wild bee diversity characteristics may, like in LU, be due to the lack of data on micro‐environmental features.

Fine‐scale structures can connect habitats, helping to counteract isolation, and thus have a positive impact on wild bee abundance and diversity by reducing flight distances between nesting and foraging sites (Steffan‐Dewenter and Tscharntke 1999). Small species with low flight capacity are especially sensitive to habitat isolation (Williams et al. 2010). However, it is known that wild bees benefit from connected resources as flight distance is directly linked with flight duration influencing the reproductive success and fitness of a species (Zurbuchen, Cheesman, et al. 2010). As foraging distances increase, nesting females have fewer brood cells, thereby decreasing the provision of pollen and nectar. As a consequence, overwintering larvae can experience a decreased survival rate due to increased parasitism (Peterson and Roitberg 2006; Seidelmann 2006).

Concluding, heterogeneity within habitats can be very helpful for wild bees. However, a recent plead urges for more heterogeneity at the landscape level (Tscharntke et al. 2025), arguing that planting annual flower strips, a short‐term local measure, can come with a series of shortcomings (also for many wild bee species) and that only heterogeneity at higher levels can sustain and restore stable populations of insect biodiversity in the long term.

### 4.2 Ecological Improvement of (Formerly) Intensively Farmed Land

The importance of high‐quality and sufficient floral supply and thus of plant choice used for ecological improvements can be best explained by study sites in LU (albeit none of the abundances of white, yellow, and blue flower colours relative to each other was returned as significant factor, Section 3.5). The investigated grasslands were characterised by rich floral resources (Table 2), although differing in previous management types (extensively or intensively farmed) and consequently recent cultivation. The former intensively‐used grasslands (IF_LU) were ecologically improved as sown wildflower fallows in 2019 and 2020 and hosted a significantly higher wild bee species richness and abundance, as well as a non‐significantly higher biomass (Figure 4). Several factors might explain the rich wild bee diversity for this habitat type: Current management practices most likely positively affected wild bee diversity as remaining plant stems provided nesting opportunities and enabled overwintering for 78% of the *Hylaeus* species detected. Such measures—leaving dead plants and debris on site—are known to be useful in restoration (see Payne et al. 2024 and references therein) and could be relatively easily applied more often. Moreover, the floral composition of cultivated plants provided high quality foraging resources for wild bees (Roulston and Goodell 2011; Scheper et al. 2015). These high‐quality flower clusters might have lured bees from further distances explaining the higher biomass detected on IF_LU sites. Furthermore, the abundant *Dipsacus fullonum* is rich in pollen and nectar and especially attracts long‐tongued bees (Cheesman 1998, Tables S1 and S2). Oligolectic pollen collectors, such as *Hoplitis adunca* (Panzer, 1798) and *Dasypoda hirtipes* (Fabricius, 1793), require specific plant species for foraging (e.g., *H. adunca* : *Echium vulgare* ; *D. hirtipes* : *Cichorium intybus* and other Cichorioideae and, to a lesser extent, Carduoideae; Westrich 2018, Westrich 2025) and were only found on ecologically improved sites (IF_LU; but also IF_AT). IF_LU sites hosted higher numbers of oligolectic species than EF_LU sites, albeit not significantly so, and plant preferences play a key role in determining the distribution of specialised species in different habitat types (Zurbuchen and Müller 2012). Finally, the increased presence of blue flowers might also explain the rich diversity of wild bees as several blue plant species (e.g., in the genera *Echium*, *Campanula*, *Vicia*) are preferred as nectar and pollen sources (Camargo et al. 2019). Generally, these results are in line with several other studies that have shown the positive effect of ecological improvement measures—if implemented thoughtfully—already after a few years and also on a landscape scale (Kulow et al. 2025; Czechofsky et al. 2025; Payne et al. 2024 and references therein).

As visible in the LU data, opportunistic wild bees can benefit from both habitat types, grassland (EF_LU) and ecologically improved formerly intensively farmed land (IF_LU)—we collected a similar composition of dominant species in both according to the rank abundance analysis. Species with high individual numbers (e.g., *L. malachurum, A. flavipes*, *A. cineraria* ) are generalists, adaptable to diverse soil compositions and foraging resources allowing them to thrive in various landscapes (Westrich 2018), and in our study, characterise both IF_LU and EF_LU communities. The conservation value of the long‐term protected EF_LU habitats, probably shaped by the gradual adaptation of different plant species influenced by low‐intensity mowing, nutrient availability, and ground structure, grounds, among others, in the slightly higher fractions of parasitic wild bee species (Section 3.4), indicating stable wild bee populations on these protected sites (Sheffield et al. 2013).

In AT, the situation differed—we found significantly higher species richness and abundance in extensively used grassland (EF_AT; Section 3.3) than in ecologically improved formerly intensively farmed land (IF_AT). While only 13 wild bee species were found to be exclusive for IF_AT habitats, this number was nearly three times higher for EF_AT habitats (Figure 4), albeit the guild of oligolectic species was not more species rich in the latter, in contrast to our initial expectation. Thus, in AT, while higher heterogeneity providing nesting and foraging possibilities positively affected wild bee communities (see Section 4.1), the overall increased abundance of floral resources on ecologically improved sites seems to have less influence. We assume that, on the one hand, EF_AT was in an ecologically better situation than EF_LU and, on the other hand, the measurement taken in IF_LU (wildflower fallows for several years) was more effective than that in IF_AT (wildflower strips) (but see Conclusion and outlook about the need to address the age of wildflower strips and fallows in the future). In line with previous studies (Roulston and Goodell 2011; Klaus et al. 2021), this might indicate that qualitatively rich supply of diverse foraging plants can be more important than the sheer quantity of supply and that aspects like synchrony between plants and wild bees—not assessed in our study—might also play important roles (Payne et al. 2024).

Taken together, our results for the two regions highlight the conservation value of both extensively farmed grassland (particularly in AT) and well‐achieved ecologically improved formerly intensively farmed land (particularly in LU), both promoting diverse and highly non‐overlapping wild bee communities (51% of species exclusively occur in only one of the habitat types in LU; 45% in AT; Figure 3).

### 4.3 Conservation Measurements in Agriculturally Dominated Land

Conservation of wild bees is a complex and multifaceted issue, and environmental measures must be carefully chosen to be successful. Our research findings emphasise the importance of extensively farmed stable grasslands (AT) as well as of biodiversity enhancement of formerly intensively farmed land (LU) for wild bee communities. In more detail, on the one hand, to sustain wild bee diversity and counteract their worldwide decline, maintaining and protecting valuable landscapes must be given a priority. Conservation efforts often also have a political dimension; for this reason, it is advisable to include independently funded, non‐profit‐oriented organisations to maintain stable, high‐priority grassland ecosystems (Zimmermann et al. 2023).

On the other hand, our study also indicates that the ecological transformation of formerly intensively used agricultural land can form valuable habitats for wild bees, especially if implemented like in IF_LU habitats. Besides floral abundance, plant diversity enhances foraging opportunities by securing a stable diet during the whole vegetation period (Ziaja et al. 2018). The diverse pool of plant species cultivated by using regionally adapted and produced seed mixtures, open soil patches, and unmown vegetation, such as on wildflower fallows in LU (i.e., IF_LU habitats), sustained diverse wild bee populations distinct from those on protected lowland hay meadows (i.e., EF_LU habitats).

Herbaceous plant species can immediately benefit wild bees, while early blooming trees and shrubs need time to develop their potential as additional pollen supply. Stable foraging resources are especially important for oligolectic bees depending on a narrow range of floral resources. Highly specialised species are most sensitive to land‐use changes, and conservation efforts should take them into account rather than relying on resilient generalists (Nieto et al. 2014; Kratschmer et al. 2021). The availability of specific floral resources can be increased through sowing of regionally tailored seed mixtures, such as those from the ‘Wëllplanzesom Lëtzebuerg’ project co‐developed by SICONA, the MNHNL and Rieger‐Hoffmann GmbH since 2018, which cultivates over 70 annual plants from local cultivars. As such, pollen and nest‐building materials for nest specialists and oligolectic wild bees can be enhanced using mixtures with key herbaceous plants such as *Papaver rhoeas* for *Hoplitis papaveris* (Latreille, 1799), *Malva moschata* and *Malva sylvestris* for *Megachile argentata* (Fabricius, 1793), and *Knautia arvensis* and other Dipsacaceae for *Andrena marginata* Fabricius, 1777.

It must be highlighted that the specialisation of wild bees includes not only pollen foraging but also the selection of nest‐building materials and suitable nesting sites. Nesting resources, and their close spatial link to available floral resources, are critical to conserve wild bees, yet they are still less understood and often overlooked in habitat restoration (Albrecht et al. 2021). Open soil patches are essential for below‐ground nesting bees; however, the specific soil microhabitat conditions essential for the majority of all wild bees are still less understood (Hall et al. 2019). While below‐ground nesting species make up almost three quarters of European bees (Zurbuchen and Müller 2012), the remaining species depend on other environmental structures, such as cavities, plant stems, and deadwood. Our data showed that conservative mowing techniques with overwintering plant stems provided stem‐nesters, such as species of the genus *Hylaeus*, opportunities to populate ecologically improved habitats in great species numbers. In addition to large‐scale, low‐intensity mowing, small‐scale, staggered or mosaic mowing can significantly improve habitat quality by leaving uncut patches—ideally relocated annually—to ensure a continuous supply of diverse foraging and nesting resources (Buri et al. 2014; Milić et al. 2024). Overall, a science‐informed, adaptive management approach is necessary and essential for wild bee conservation, integrating updated research (e.g., Payne et al. 2024 and references therein) and the principles of the EU Biodiversity Strategy 2030 (European Commission 2020). National pollinator action plans are an important implementation tool here, accounting for national legislation and regional settings (e.g., geography). For example, the Luxembourg National Action Plan for Pollinators (MECDD 2021) mandates species‐specific measures for threatened wild bees (Action 10) and their implementation into adaptive management regimes across habitat types (Actions 3–6). As a result, and based on the conservation action plan for Teasel‐plant specialised bees in Europe (Michez et al. 2023), a National Action Plan for *Andrena hattorfiana* (Fabricius, 1755) was developed and is getting implemented (Andrási et al. 2024). In parallel, the currently developed National BuzzLine Action Plan identifies key pollinator areas and connectivity corridors for future pollinator‐friendly habitat management and restoration (B. Andrási, pers. comm.) following recommendations of Deiss et al. (2026).

### 4.4 Conclusion and Outlook

Our study corroborates that habitats characterised by a high degree of heterogeneity (e.g., ecotones and patchiness) and abundant floral resources can offer refuge to both wild bee generalists and specialists—but it is these ecological specialists that are particularly threatened by habitat loss and degradation (Kratschmer et al. 2021; Michez et al. 2026). In more detail, we detected opposed effects when comparing ecologically improved grasslands in agriculture and long‐term stable landscapes in two Central European areas. The extensively farmed grasslands (EF_AT) in AT were higher in wild bee species richness and abundance than ecologically improved formerly intensively farmed land (IF_AT). Likely, the wood‐ecotones of EF_AT were the primary driver explaining the differences in species communities. Considering the various factors, including management system, structure diversity, and nesting opportunities, for AT we can conclude that wild bee abundance was linked to richly structured habitat patches serving as nesting and foraging sites.

However, ecological improvement of sites in an agricultural context can positively impact wild bee diversity by providing high‐quality and abundant habitat with (nearby) floral resources supporting foraging and nest construction. This was shown for the formerly intensively used and recently ecologically improved land (IF_LU) in LU, but long‐term protected, extensively used grasslands in the same area cannot be compensated for owed to their unique bee species composition. Our results demonstrate that the efficacy of habitat heterogeneity and ecological improvements is highly context‐dependent, likely mediated by regional landscape matrices and specific microenvironmental resources.

Future studies on the relative value for wild bees of conservation strategies in grassland of Austria and Luxembourg and beyond should consider, among others, (i) longer‐term monitoring to evaluate the effectiveness of measures such as wildflower strips and fallows, considering that, for example, the positive impact of flower abundance and plant species richness on wild bee diversity may significantly decrease after the initial years (Albrecht et al. 2021); (ii) quantifying microenvironmental habitat features of each study site, such as the proportion of open soil and the quantity of plant stems of various types, which are not accessible via GIS analysis, for a potentially more robust analysis of the factors beneficial to wild bees than accessible via ecotone quantification; (iii) assessing both the species diversity and quality (pollen, nectar) of flowers as likely crucial parameters to wild bee populations (Payne et al. 2024)—together with quantifying flower‐colour diversity as done in this study, this will allow evaluating the relevance of the latter to pollinators (Xie et al. 2025) relative to that of the former two; in assessing flower species diversity, also considering the synchrony between plant and wild bee species will be important (Payne et al. 2024 and references therein); (iv) using at least one additional, complementary method of sampling wild bees, such as vane traps, Malaise traps or direct observation, to reduce the potential biases of any single method used, such as that of pan traps in this study, on species richness (Anderson et al. 2024) as well as abundance inferred biomass data (Mathis et al. 2024); and (v) assessing the load of two types of chemical pollutants in the habitats that are known to affect wild bees, that is, pesticides (Dicks et al. 2021) and forever chemicals (Hierlmeier‐Hackl et al. 2026).

## Author Contributions

**Julia Lanner:** formal analysis (lead), writing – original draft (equal). **Pauline Bühler:** conceptualization (supporting), formal analysis (supporting), investigation (equal), writing – original draft (equal), writing – review and editing (supporting). **Loïc Hellers:** conceptualization (supporting), formal analysis (supporting), investigation (equal), writing – original draft (equal), writing – review and editing (supporting). **Timotheus Kopf:** conceptualization (supporting), investigation (supporting), writing – review and editing (supporting). **Alexander Weigand:** conceptualization (equal), supervision (equal), writing – review and editing (equal). **Birgit C. Schlick‐Steiner:** conceptualization (equal), formal analysis (supporting), supervision (equal), writing – review and editing (equal). **Florian M. Steiner:** conceptualization (equal), formal analysis (supporting), supervision (equal), writing – review and editing (equal).

## Funding

Universität Innsbruck supported P.B. and L.H. financially. The National Museum of Natural History Luxembourg financially supported fieldwork activities and DNA barcoding.

## Conflicts of Interest

The authors declare no conflicts of interest.

## Supporting information


**Data S1:** Specimens for which both a morphological and a genetic identification (applying the protocol in Weigand, A.M., Herrera‐Mesías, F. (2020). First record of the wild bee Eucera (Tetralonia) alticincta (Lepeletier, 1841) in Luxembourg. *Bulletin de la Société des Naturalistes Luxembourgeois*, 122, 141–146) were performed. The final taxonomic assignment for each specimen is underlined.

**Data S2:** Abundance, species richness, evenness, Shannon index, biomass, number of polygons (polygon), lengths of ecotones involving single trees, bushes, hedgerows or woods for trees and bushes (eco tree) and of ecotones not involving these (eco fields), as well as flower coverage of white, yellow and blue colour for all sites analysed. AT, Austria; EF, extensively used grassland; IF, ecologically improved formerly intensively farmed land; LU, Luxembourg.

**Data S3:** Number of polylectic, oligolectic, below‐ground nesting, above‐ground nesting, solitary, social and parasitic wild bee species for all sites analysed. AT, Austria; EF, extensively used grassland; IF, ecologically improved formerly intensively farmed land; LU, Luxembourg.

**Data S4:** Total abundance; total biomass; abundance of below‐ground nesting, above‐ground nesting, oligolectic, polylectic, social, solitary and parasitic wild bee species, as well as environmental factors (number of polygons [polygon], lengths of ecotones involving single trees, bushes, hedgerows or woods for trees and bushes [eco tree] and of ecotones not involving these [eco fields], as well as flower coverage of white, yellow and blue colour) on all sites studied in Austria. Group 1—extensively used grassland; Group 2—ecologically improved formerly intensively farmed land.

**Data S5:** Total abundance; total biomass; abundance of below‐ground nesting, above‐ground nesting, oligolectic, polylectic, social, solitary and parasitic wild bee species, as well as environmental factors (number of polygons [polygon], lengths of ecotones involving single trees, bushes, hedgerows or woods for trees and bushes [eco tree] and of ecotones not involving these [eco fields], as well as flower coverage of white, yellow and blue colour) on all sites studied in Luxembourg. Group 1—ecologically improved formerly intensively farmed land; Group 2—extensively used grassland.

**Figure S1:** The abundance, species richness and ecological guilds of wild bees were studied in Central European grassland meadows located in Austria (EF_AT, IF_AT) and Luxembourg (EF_LU, IF_LU). The habitats were classified according to their management intensity; EF, extensively used grassland; IF, ecologically improved formerly intensively farmed land.
**Figure S2:** Wild bees were sampled passively using coloured pan traps. In total, we placed 126 pan trap sets across 28 study sites for 4 months and replaced the traps approximately every 10 days. To prevent interferences with ravens and wild boars, we added nails on top of the wooden sticks and mounted green robes thighs filled with dog hair on intensively farmed (IF) sites, respectively.
**Figure S3:** Development of wild bee and honeybee abundances summed over all traps across the eight sampling periods (T1–T8) in the investigated habitats in Luxembourg (EF_LU, IF_LU) and Austria (EF_AT, IF_AT). EF, extensively used grassland; IF, ecologically improved formerly intensively farmed land.
**Figure S4:** Boxplots illustrate significant differences in the Shannon index and evenness between EF_AT and IF_AT habitats in Austria. In contrast, the investigated habitats in Luxembourg (EF_LU, IF_LU) had similar index values. EF, extensively used grassland; IF, ecologically improved formerly intensively farmed land.
**Figure S5:** The composition of wild bee communities, as characterised by their species‐specific life history traits (a, b nesting specialisations; c, d dietary specialisations; e, f sociality), differed significantly in all traits between EF_LU and IF_LU habitats except oligolectic pollen selection (c); conversely, EF_AT and IF_AT habitats had more similar wild‐bee communities, except for nesting above‐ground (b) polylectic pollen selection (d), and solitary lifestyle (f). EF, extensively used grassland; IF, ecologically improved formerly intensively farmed land.
**Table S1:** Seed mixture ‘LUX‐Buntbrache’ sowed on formerly intensively farmed, ecologically improved (IF_LU) grasslands in Luxembourg from Rieger‐Hoffmann Gmbh company.
**Table S2:** The extensively farmed (EF_LU) and the formerly intensively farmed, ecologically improved (IF_LU) study sites located in Luxembourg were both characterised by abundant floral resources providing foraging opportunities for bees, as reflected in the list of the identified dominant flower species that bloomed between April and July.
**Table S3:** Numbers of individuals for all bee species sampled in Austria, per site, per habitat type and in total; EF, extensively used grassland, IF = ecologically improved formerly intensively farmed land.
**Table S4:** Numbers of individuals for all bee species sampled in Luxembourg, per site, per habitat type and in total; EF, extensively used grassland; IF, ecologically improved formerly intensively farmed land.
**Table S5:** Compiled list of species numbers for the evaluated species‐specific ecological traits of wild bees collected in Austria (AU; RS‐EF, richly‐structured and extensively farmed; PS‐IF, poorly‐structured and intensively farmed) and Luxembourg (LU; EF, extensively farmed; IF, intensively farmed).

## Data Availability

The datasets analysed during the current study are included in the Supplement as Data S1 to S5.
