# Supplementary material for: Wild Bee Conservation: Habitat Heterogeneity and Ecological Improvement in Agricultural Land With Open Grassland
Source: Ecol Evol. 2026 Oct 16;16(10):e74514. doi: 10.1002/ece3.74514 (PMC13675122; doi:10.1002/ece3.74514)
Supplement: Supplementary file 1 [file ECE3-16-e74514-s002.docx]

Supplementary Material for

**Wild bee conservation: habitat heterogeneity and ecological improvement in agricultural land with open grassland**

Julia Lanner, Pauline Bühler, Loïc Hellers, Timotheus Kopf , Alexander Weigand, Birgit C. Schlick-Steiner, Florian M. Steiner

Figure S1: The abundance, species richness, and ecological guilds of wild bees were studied in Central European grassland meadows located in Austria (EF_AT, IF_AT) and Luxembourg (EF_LU, IF_LU). The habitats were classified according to their management intensity;EF = extensively used grassland; IF = ecologically improved formerly intensively farmed land.

Figure S2: Wild bees were sampled passively using coloured pan traps. In total, we placed 126 pan trap sets across 28 study sites for four months and replaced the traps approximately every ten days. To prevent interferences with ravens and wild boars, we added nails on top of the wooden sticks and mounted green robes thighs filled with dog hair on intensively farmed (IF) sites, respectively.

Figure S3. Development of wild bee and honeybee abundances summed over all traps across the eight sampling periods (T1-T8) in the investigated habitats in Luxembourg (EF_LU, IF_LU) and Austria (EF_AT, IF_AT). EF = extensively used grassland; IF = ecologically improved formerly intensively farmed land.

Figure S4: Boxplots illustrate significant differences in the Shannon index and evenness between EF_AT and IF_AT habitats in Austria. In contrast, the investigated habitats in Luxembourg (EF_LU, IF_LU) had similar index values. EF = extensively used grassland; IF = ecologically improved formerly intensively farmed land.

Figure S5: The composition of wild bee communities, as characterized by their species-specific life history traits (a, b nesting specializations; c, d dietary specializations; e, f sociality), differed significantly in all traits between EF_LU and IF_LU habitats except oligolectic pollen selection (c); conversely, EF_AT and IF_AT habitats had more similar wild-bee communities, except for nesting above-ground (b) polylectic pollen selection (d), and solitary lifestyle (f). EF = extensively used grassland, IF = ecologically improved formerly intensively farmed land.

Table S1: Seed mixture “LUX-Buntbrache” sowed on formerly intensively farmed, ecologically improved (IF_LU) grasslands in Luxembourg from Rieger-Hoffmann Gmbh company.

| **wild flowers 40%** | **arable crops 60%** |
| --- | --- |
| *Anthemis tinctoria* | *Brassica oleracea* |
| *Arctium lappa* | *Brassica rapa* |
| *Barbarea vulgaris* s.str. | *Fagopyrum esculentum* |
| *Centaurea cyanus* | *Foeniculum vulgare* |
| *Cichorium intybus* | *Helianthus annuus* 'Peredovick' |
| *Daucus carota* | *Lepidium sativum* |
| *Dipsacus fullonum* | *Linum usitatissimum* |
| *Hypericum perforatum* | *Medicago sativa* |
| *Isatis tinctoria* | *Nigella sativa* |
| *Linaria vulgaris* | *Petroselinum crispum* 'Mooskrause' |
| *Malva moschata* | *Raphanus sativus* |
| *Malva sylvestris*  s.str. | *Spinaca oleracea* 'Matador' |
| *Melilotus albus* | *Vicia sativa* |
| *Oenothera biennis* agg. |  |
| *Origanum vulgare* |  |
| *Papaver rhoeas* |  |
| *Pastinaca sativa* s.str. |  |
| *Reseda luteola* |  |
| *Saponaria officinalis* |  |
| *Silene dioica* |  |
| *Silene latifolia alba* |  |
| *Verbascum lychnitis* |  |
| *Verbascum nigrum* |  |
| *Verbascum thapsus* |  |
| *Verbena officinalis* |  |

Table S2: The extensively farmed (EF_LU) and the formerly intensively farmed, ecologically improved (IF_LU) study sites located in Luxembourg were both characterized by abundant floral resources providing foraging opportunities for bees, as reflected in the list of the identified dominant flower species that bloomed between April and July.

| EF | IF |
| --- | --- |
| *Ajuga reptans* | [*Centaurea cyanus*](https://www.inaturalist.org/observations/102162583) |
| *Alopecurus pratensis* |  |
| *Centaurea jacea* | [*Dipsacus fullonum*](https://www.inaturalist.org/observations/102162593) |
| *Erophila verna* | [*Echium vulgare*](https://www.inaturalist.org/observations/102162586) |
| *Gardamine pratensis* | [*Lamium purpureum*](https://www.inaturalist.org/taxa/55851) |
| *Lathyrus tuberosus* |  |
| *Lotus corniculatus* | [*Linaria vulgaris*](https://www.inaturalist.org/observations/102162597) |
|  | [*Malva moschata*](https://www.inaturalist.org/observations/102162590) |
| *Lynchis flos-cuculi* | [*Melilotus albus*](https://www.inaturalist.org/observations/102162591) |
| *Myosotis arvensis* | [*Securigera varia*](https://www.inaturalist.org/observations/102162596) |
| *Primula veris* | [*Senecio vulgaris*](https://www.inaturalist.org/taxa/53211) |
| *Ranunculus bulbosus* |  |
| *Saxifraga granulata* | [*Silene dioica*](https://www.inaturalist.org/taxa/55759) |
|  | [*Silene latifolia*](https://www.inaturalist.org/observations/102162574) |
| *Senecio jacobaea* | *Trifolium repens* |
| *Stellaria graminea* | [*Veronica persica*](https://www.inaturalist.org/taxa/55666) |
| *Taraxacum* | *Viola arvensis* |
| *Trifolium pratensis* |  |

Table S3. Numbers of individuals for all bee species sampled in Austria, per site, per habitat type, and in total; EF = extensively used grassland, IF = ecologically improved formerly intensively farmed land.

Table S4. Numbers of individuals for all bee species sampled in Luxembourg, per site, per habitat type, and in total; EF = extensively used grassland, IF = ecologically improved formerly intensively farmed land.

Table S5: Compiled list of species numbers for the evaluated species-specific ecological traits of wild bees collected in Austria (AU; RS-EF = richly-structured and extensively farmed, PS-IF = poorly-structured and intensively farmed) and Luxembourg (LU; EF = extensively farmed and IF = intensively farmed).

|  |  |  | **dietary specialisation** | | **nesting specialisation** | | **sociality** | | |
| --- | --- | --- | --- | --- | --- | --- | --- | --- | --- |
| **country** | **habitat type** | **site** | **oligolectic** | **polylectic** | **below-ground** | **above-ground** | **solitary** | **social** | **parasitic** |
| AU | EF_AT | RS-EF1 | 4 | 23 | 22 | 7 | 16 | 11 | 1 |
| AU | EF_AT | RS-EF2 | 5 | 31 | 24 | 15 | 23 | 10 | 2 |
| AU | EF_AT | RS-EF3 | 2 | 27 | 21 | 11 | 18 | 9 | 2 |
| AU | EF_AT | RS-EF4 | 4 | 30 | 24 | 14 | 19 | 12 | 6 |
| AU | EF_AT | RS-EF5 | 6 | 29 | 32 | 7 | 21 | 12 | 3 |
| AU | EF_AT | RS-EF6 | 4 | 31 | 29 | 10 | 16 | 14 | 1 |
| AU | EF_AT | RS-EF7 | 4 | 30 | 27 | 10 | 21 | 11 | 2 |
| AU | IF_AT | PS-IF8 | 3 | 23 | 25 | 4 | 16 | 8 | 0 |
| AU | IF_AT | PS-IF9 | 3 | 14 | 14 | 6 | 10 | 6 | 1 |
| AU | IF_AT | PS-IF10 | 4 | 18 | 20 | 5 | 13 | 9 | 1 |
| AU | IF_AT | PS-IF11 | 7 | 20 | 23 | 5 | 18 | 7 | 1 |
| AU | IF_AT | PS-IF12 | 5 | 27 | 29 | 7 | 18 | 13 | 4 |
| AU | IF_AT | PS-IF13 | 4 | 17 | 18 | 4 | 12 | 9 | 1 |
| AU | IF_AT | PS-IF14 | 7 | 24 | 26 | 10 | 15 | 14 | 4 |
| LU | EF_LU | BW1 | 3 | 23 | 23 | 6 | 18 | 8 | 2 |
| LU | EF_LU | BW2 | 6 | 30 | 33 | 6 | 26 | 10 | 7 |
| LU | EF_LU | BW3 | 1 | 17 | 18 | 3 | 11 | 7 | 2 |
| LU | EF_LU | BW4 | 2 | 25 | 23 | 8 | 16 | 11 | 2 |
| LU | EF_LU | BW5 | 1 | 27 | 27 | 4 | 20 | 8 | 0 |
| LU | EF_LU | BW6 | 2 | 22 | 19 | 6 | 17 | 7 | 4 |
| LU | EF_LU | BW7 | 1 | 25 | 20 | 8 | 15 | 11 | 2 |
| LU | IF_LU | BU1 | 4 | 27 | 26 | 7 | 22 | 9 | 2 |
| LU | IF_LU | BU2 | 6 | 32 | 27 | 13 | 27 | 11 | 2 |
| LU | IF_LU | BU3 | 3 | 38 | 36 | 9 | 23 | 18 | 3 |
| LU | IF_LU | BU4 | 2 | 33 | 28 | 10 | 22 | 13 | 0 |
| LU | IF_LU | BU5 | 5 | 43 | 38 | 14 | 32 | 16 | 0 |
| LU | IF_LU | BU6 | 3 | 34 | 33 | 6 | 25 | 12 | 2 |
| LU | IF_LU | BU7 | 1 | 29 | 26 | 6 | 21 | 9 | 6 |

Data S1: Specimens for which both a morphological and a genetic identification (applying the protocol in Weigand, A.M., Herrera-Mesías, F. (2020) First record of the wild bee Eucera (Tetralonia) alticincta (Lepeletier, 1841) in Luxembourg. Bulletin de la Société des Naturalistes Luxembourgeois, 122, 141–146) were performed. The final taxonomic assignment for each specimen is underlined. Accessible as standalone file “DataS1.xlsx”.

Data S2: Abundance, species richness, evenness, Shannon index, biomass, number of polygons (polygon), lengths of ecotones involving single trees, bushes, hedgerows, or woods for trees and bushes (eco tree) and of ecotones not involving these (eco fields), as well as flower coverage of white, yellow, and blue colour for all sites analysed. AT = Austria, LU = Luxembourg, EF = extensively used grassland, IF = ecologically improved formerly intensively farmed land. Accessible as standalone file “DataS2.xlsx”.

Data S3: Number of polylectic, oligolectic, below-ground nesting, above-ground nesting, solitary, social, and parasitic wild bee species for all sites analysed. AT = Austria, LU = Luxembourg, EF = extensively used grassland, IF = ecologically improved formerly intensively farmed land. Accessible as standalone file “DataS3.xlsx”.

Data S4: Total abundance; total biomass; abundance of below-ground nesting, above-ground nesting, oligolectic, polylectic, social, solitary, and parasitic wild bee species, as well as environmental factors (number of polygons (polygon), lengths of ecotones involving single trees, bushes, hedgerows, or woods for trees and bushes (eco tree) and of ecotones not involving these (eco fields), as well as flower coverage of white, yellow, and blue colour) on all sites studied in Austria. Group 1 = extensively used grassland, group 2 = ecologically improved formerly intensively farmed land. Accessible as standalone file “DataS4.xlsx”.

Data S5: Total abundance; total biomass; abundance of below-ground nesting, above-ground nesting, oligolectic, polylectic, social, solitary, and parasitic wild bee species, as well as environmental factors (number of polygons (polygon), lengths of ecotones involving single trees, bushes, hedgerows, or woods for trees and bushes (eco tree) and of ecotones not involving these (eco fields), as well as flower coverage of white, yellow, and blue colour) on all sites studied in Luxembourg. Group 1 = ecologically improved formerly intensively farmed land, group 2 = extensively used grassland. Accessible as standalone file “DataS5.xlsx”.
